# HBV p22-interacting protein C1QBP inhibits viral replication through impeding nucleocapsid formation and nuclear import

**DOI:** 10.1371/journal.ppat.1013581

**Published:** 2025-10-17

**Authors:** Xiao Peng, Cheng-Der Liu, Bidisha Mitra, Elena S. Kim, Ning Sun, Andrea Jurado, Hu Zhang, Shitao Li, Tongqing Zhou, Haitao Guo

**Affiliations:** 1 Department of Microbiology and Molecular Genetics; Cancer Virology Program, UPMC Hillman Cancer Center, University of Pittsburgh School of Medicine, Pittsburgh, Pennsylvania, United States of America; 2 School of Medicine, Tsinghua University, Beijing, China; 3 Department of Microbiology and Immunology, Tulane University, New Orleans, Louisiana, United States of America; 4 Vaccine Research Center, National Institute of Allergy and Infectious Diseases, National Institutes of Health, Bethesda, Maryland, United States of America; Pennsylvania State University College of Medicine: Penn State College of Medicine, UNITED STATES OF AMERICA

## Abstract

The circulating hepatitis B virus (HBV) e antigen (HBeAg) is known to subvert the host immune system to benefit chronic HBV infection. However, the biological function of a major intracellular form of HBeAg, specifically the precore protein intermediate of 22 kDa (p22) lacking the N-terminal signal peptide, remains largely unclear. Through pull-down and mass spectrometry analysis, we re-identified the complement C1q binding protein (C1QBP) as a p22-binding protein. Immunofluorescence results demonstrated that C1QBP was predominantly localized in the mitochondrial matrix and the leaked C1QBP interacted with p22 in the cytosol. Using co-immunoprecipitation assay, we mapped the arginine-rich, highly positively charged C-terminal domain (CTD) of p22 and the internal domain aa 74–160 of C1QBP as binding domains for p22-C1QBP interaction. By studying the impact of C1QBP on HBV replication, we found that C1QBP overexpression led to the autolysosomal degradation of HBV core protein (HBc) and significantly reduced viral nucleocapsid formation in a p22-dependent manner. Additionally, a C1QBP mutant without the mitochondrial targeting signal (MTS) exhibited a greater inhibitory effect on HBV replication compared to the wild type (wt). Although HBc and p22 share the same CTD sequence, C1QBP does not bind to wt HBV capsid. However, disrupting capsid assembly by HBc-Y132A mutant or CAM-A (class A capsid assembly modulator) treatment enables HBc-C1QBP interaction. Moreover, C1QBP binds to the CTD of HBc on the cytoplasmic deproteinated relaxed circular DNA (DP-rcDNA)-containing capsid that is partially disassembled, hindering DP-rcDNA nuclear import and subsequent covalently closed circular DNA (cccDNA) formation. Collectively, our study suggests that C1QBP inhibits HBV replication through dual mechanisms, proposing a novel therapeutic approach for managing chronic HBV infection.

## Author summary

Hepatitis B virus (HBV) remains a major global public health burden, causing chronic liver infections that can lead to severe diseases such as cirrhosis and liver cancer. HBV produces only seven proteins, among which the biological function of a major intracellular precursor of the circulating viral e antigen (HBeAg), namely p22, remains poorly understood. In our study, we re-identified a host protein, the complement C1q binding protein (C1QBP) leaked from mitochondria, as an intracellular binding partner of p22. Further investigation revealed two mechanisms by which C1QBP regulates HBV replication: first, C1QBP promotes the autolysosomal degradation of the viral core protein in a p22-dependent manner; second, C1QBP blocks the nuclear import of nucleocapsid by competitively binding to the nuclear localization signal of viral capsid protein exposed on the nucleocapsid containing deproteinated mature viral DNA genome, subsequently reducing the production of viral cccDNA in the nucleus. Our findings not only shed light on the virus-host interaction between p22 and C1QBP but also indicate the potential of C1QBP as an antiviral strategy for treating hepatitis B.

## Introduction

Human hepatitis B virus (HBV) is a hepatotropic DNA virus belonging to the *Hepadnaviridae* family, leading to both acute and chronic liver diseases upon infection [1,2]. Approximately 257 million individuals are living with chronic HBV infection, with an estimated 1 million deaths reported annually, primarily due to complications such as cirrhosis and hepatocellular carcinoma [3].

HBV infects hepatocyte via attaching to its receptor, namely the sodium taurocholate co-transporting polypeptide (NTCP), on the cell surface [4]. Once entering the cell through endocytosis, the viral capsid delivers the relaxed circular DNA (rcDNA) genome to the nucleus, where the rcDNA is converted into covalently closed circular DNA (cccDNA) [5]. HBV cccDNA exists as an episomal minichromosome, serving as the template for the transcription of viral RNAs, including the 3.5 kb precore mRNA that encodes the precore protein (p25), 3.5 kb pregenomic RNA (pgRNA) for the core protein (HBc, p21) and the polymerase (Pol), 2.4 kb mRNA for the large (L) surface protein (HBs), 2.1 kb mRNA for the middle (M) and small (S) surface proteins, and 0.7 kb mRNA for the X protein (HBx) [1,2]. The pgRNA is then encapsidated by HBc and retro-transcribed by HBV Pol to form rcDNA within nucleocapsids. The mature nucleocapsid is either redirected to the nucleus to replenish the cccDNA pool via the recycling pathway or enveloped and secreted as a progeny virion [6].

The precore mRNA translates the precore protein, a 25 kDa precursor that is ultimately processed to form the 17 kDa secreted hepatitis B e antigen (HBeAg) [1]. HBeAg exists as a circulating nonstructural protein that does not directly participate in viral replication [7]. Clinically, HBeAg serves as an important biomarker for assessing the phase of chronic HBV infection. Patients positive for HBeAg typically display elevated viral replication and higher infectivity compared to HBeAg-negative patients [8]. Loss of HBeAg accompanied by seroconversion to anti-HBe antibodies, generally indicates a favorable therapeutic response and improved prognosis in individuals with chronic hepatitis B (CHB) [9].

The precore/core open reading frame (ORF) contains two distinct in-frame AUG start codons: the upstream AUG initiates translation of the precore protein from precore mRNA, while the downstream AUG initiates translation of the core protein from pgRNA [2]. Structurally, core proteins can spontaneously assemble into viral capsid upon being translated in the cytoplasm. On the other hand, precore/p25 differs from core protein by featuring a unique 29 amino acid (aa) N-terminal extension. The initially translated 19 aa signal peptide is recognized by the cellular signal recognition particle (SRP) complex, guiding the precore mRNA template to the endoplasmic-reticulum (ER) membrane, where the signal peptide is co-translationally cleaved off, resulting in p22. This p22 intermediate is either translocated into the ER/Golgi or released back to the cytosol [10]. In the Golgi lumen, the arginine-rich C-terminal domain (CTD) of p22 is cleaved by furin endopeptidase to release HBeAg into the extracellular milieu in an antiparallel homodimeric form [11,12]. Due to the presence of nuclear localization signal (NLS) on p22, this protein can also be transported into the nucleus [13]. The biological functions of p22 in the cytosol and nucleus remain largely unknown.

The complement C1q binding protein (C1QBP, also known as gC1qR or p32), initially identified as a binding partner for the globular head region of the first component of the complement system (C1q) [14], is a multifunctional protein involved in diverse cellular and immunological processes. The C1QBP monomer is comprised of 282 aa residues, with the N-terminal aa 1–73 forming a mitochondrial targeting signal (MTS) responsible for directing protein to mitochondrial matrix, where it plays critical roles in Ca^2+^ uptake and mitochondrial oxidative phosphorylation (OXPHOS) [15–19]. Subsequently, the MTS is cleaved off to generate the mature form of C1QBP, which assembles into a donut-shaped homotrimer predominantly located in the mitochondrial matrix but is also distributed in other subcellular compartments, including cytosol, plasma membrane, and nucleus [20–23]. C1QBP is known to bind to human complement subcomponent C1q molecules and inhibit C1 activation [23]. In the nucleus, it acts as a transcriptional suppressor, notably inhibiting FOXC1-mediated transcriptional activation [24–26]. Recent studies have demonstrated that C1QBP negatively regulates cellular innate immune responses. It interacts with mitochondrial antiviral-signaling protein MAVS, suppressing downstream type I interferon (IFN) signaling cascades mediated by RIG-I and MDA5 [27]. The cytosolic C1QBP binds to and inhibits cyclic GMP-AMP synthase (cGAS), thereby attenuating cGAMP production and subsequent IFN-I signaling [28]. Moreover, C1QBP has been shown to interact with viral proteins from various pathogens, including Epstein-Barr Virus (EBV), Cytomegalovirus (CMV), Herpes Simplex Virus Type 1 (HSV-1), and rubella virus, to promote or reactivate viral replication [29–33]. In terms of HBV, C1QBP has been shown as an intracellular p22 binding partner more than 20 years ago [34,35], but the previous studies were mainly performed in non-hepatic cell lines, and the biological function of p22-C1QBP interaction was unknown.

In this study, making use of our previously established HepHA-HBe4 cell line that stably expresses HA-tagged HBV precore/HBeAg [36], we conducted an anti-HA pull-down combined with proteomic analysis, reidentifying and confirming C1QBP as a bona fide p22-binding protein. We further demonstrated that C1QBP interacts with the CTD of p22 through its internal 74–160 aa region. Functionally, we found that C1QBP negatively regulates HBV replication by promoting autolysosomal degradation of the core protein in a p22-dependent manner. In addition, C1QBP inhibits the nuclear import of deproteinated rcDNA (DP-rcDNA) through competitively interacting with the CTD of core protein on partially disassembled nucleocapsid, resulting in reduction of cccDNA formation in the nucleus. These findings revealed a previously unrecognized antiviral role of C1QBP in HBV replication and cccDNA formation, thus providing novel insights into host-virus interactions and potential therapeutic targets.

## Results

### Identification of host proteins interacting with HBV p22 by mass spectrometry

**Fig 1:**
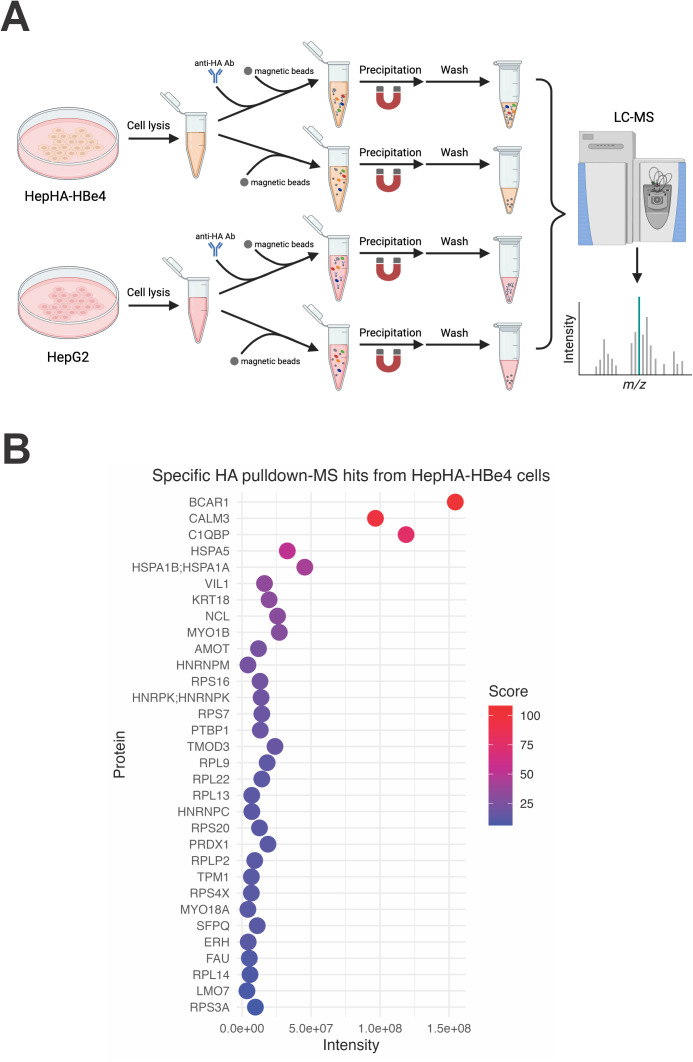
Identification of HBV p22-binding proteins. (A) Diagram of HA-p22 pull-down and mass spectrometry analysis. Cell lysates from HepHA-HBe4 and HepG2 cells were incubated with anti-HA antibody and magnetic protein G beads or beads only. After pull-down and washing, the bead-bound proteins were digested and identified by liquid chromatography-tandem mass spectrometry (LC-MS/MS). Created in BioRender (https://BioRender.com/ctrm7wi). (B) Proteins specifically enriched in the anti-HA pull-down from HepHA-HBe4 cells were ranked based on their intensities and confidence scores. Bubble plots were created using the ggplot2 package in R (version 4.2.2).

To identify host proteins that interact with HBV p22, we conducted a pull-down assay followed by liquid chromatography-tandem mass spectrometry (LC-MS/MS) analysis (Fig 1A). The previously developed stable cell line HepHA-HBe4, which constitutively expresses HA-tagged p22/HBeAg, was employed [36], while the parental HepG2 cell line served as a negative control. Cell lysates from HepHA-HBe4 and HepG2 cells were subsequently incubated with anti-HA beads or control beads, which were then precipitated. After washing and on-beads digestion, the bound peptides were sequenced by LC-MS/MS and identified using the UniProt database. Proteins identified in HepHA-HBe4 but not in HepG2 or control beads samples were ranked according to binding score and intensity, as summarized in Fig 1B. Among the p22-associated protein candidates identified, the top three hits are BCAR1 (Breast Cancer Anti-Estrogen Resistance 1), CALM3 (Calmodulin-3), and C1QBP; other proteins of interest (POIs) include ribosomal proteins, members of the heat shock protein family, and so on (Fig 1B). Notably, C1QBP was detected with high intensity and binding score, consistent with previous studies reporting its interaction with p22 [34,35]. Considering the low expression level of BCAR1 in the liver and the common occurrence of CALM3 in pull-down MS analysis, we prioritized C1QBP for further study based on its reported regulatory effects on other viruses and innate signaling [27,28,37–39].

### Validation of C1QBP as a cellular binding partner of HBV p22

**Fig 2:**
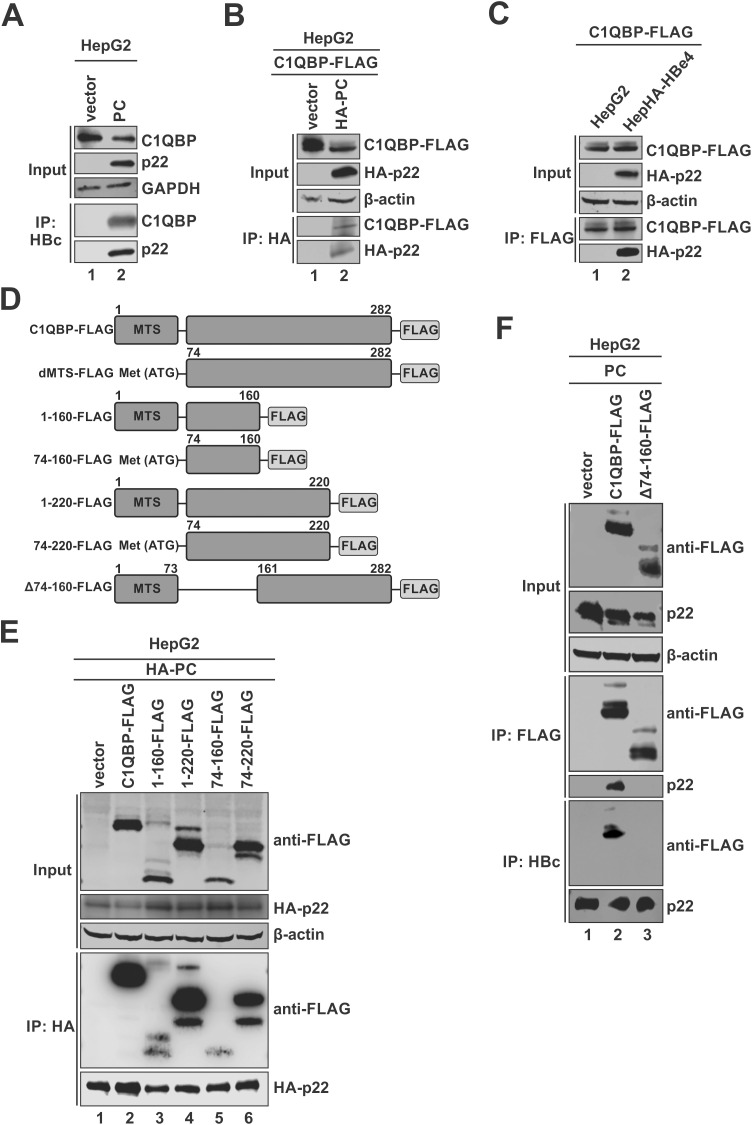
Validation of p22-C1QBP interaction and mapping the p22-interacting domain on C1QBP. (A) HepG2 cells were transfected with precore (PC) or control vector for 3 days. 10% of cells were collected and lysed as input samples, and the remaining cells were subjected to co-immunoprecipitation (co-IP) using anti-HBc antibody. The input and immunoprecipitated samples were subsequently analyzed by Western blot using antibodies against C1QBP and HBc, respectively. GAPDH was used as a loading control for input samples. (B) HepG2 cells were co-transfected with HA-tagged precore (HA-PC) or control vector together with FLAG-tagged C1QBP expression vector (C1QBP-FLAG) for 3 days. 10% of cells were collected and lysed as input samples, and the remaining cells were subjected to co-IP with anti-HA antibody. The input and immunoprecipitated samples were analyzed by Western blot using antibodies against FLAG epitope and HA epitope, respectively. β-actin served as a loading control for input samples. (C) HepG2 and HepHA-HBe4 cells were transfected with C1QBP-FLAG for 3 days. 10% of the cells were collected as input samples, and the remaining cells were subjected to anti-FLAG co-IP. Input and immunoprecipitated samples were analyzed by Western blot using anti-FLAG and anti-HA antibodies. β-actin was used as a loading control for input samples. (D) Schematic illustration of the C-terminally FLAG-tagged full-length C1QBP and deletion mutants. MTS: mitochondrial targeting signal. (E) HepG2 cells were co-transfected with HA-PC and control vector, or each indicated C1QBP-FLAG construct shown in panel D, for 3 days, followed by co-IP analyses as described in panel B. (F) HepG2 cells were co-transfected with PC and either a control vector, full-length C1QBP-FLAG, or the Δ74-160-FLAG truncation mutant. Three days post-transfection, 10% of cells were collected and lysed as input samples, and the remaining cells were subjected to reciprocal co-IP using anti-FLAG and anti-HBc antibodies. The input and co-IP samples were subsequently analyzed by Western blot. β-actin served as a loading control for input samples.

To validate the interaction between C1QBP and HBV p22 identified by anti-HA pull-down MS, we performed co-immunoprecipitation (co-IP) assay in HepG2 cells transfected with either a control vector or the precore plasmid expressing untagged p22. The results clearly demonstrated a specific interaction between the endogenous C1QBP and the wild type (wt) p22 (Fig 2A). This interaction was further confirmed in HepG2 cells overexpressing C-terminally FLAG-tagged C1QBP (C1QBP-FLAG) and HA-p22 (Fig 2B), as well as in HepHA-HBe4 cells overexpressing C1QBP-FLAG (Fig 2C).

Interestingly, the endogenous C1QBP was detected as a single band on Western blot (Fig 2A), whereas the overexpressed C1QBP-FLAG exhibited a doublet band pattern, and both forms of C1QBP-FLAG interacted with p22 (Fig 2B, C). Considering that the MTS of C1QBP is cleaved upon mitochondrial import [20], we hypothesized that the higher molecular weight band represents the immature forms of C1QBP due to incomplete MTS cleavage upon overexpression. To examine this possibility, we expressed the untagged full-length C1QBP and its MTS-deletion mutant (dMTS) in HepG2 cells, followed by subcellular fractionation analysis. As shown in S1A Fig, the endogenous C1QBP was detected in both mitochondrial and cytosolic fractions as a single band migrating at the same position as the dMTS mutant (lanes 1 and 3). The weak signal of C1QBP detected in the mitochondrial fraction of cells overexpressing dMTS was attributed to the endogenous C1QBP (lane 3). Conversely, the overexpressed full-length C1QBP exhibited multiple bands, including the lower band migrating similarly to endogenous C1QBP, the middle band corresponding to the uncleaved full-length form, and an upper band with higher molecular weight (lane 2). This upper band was not consistently observed across experiments, and it appeared to be associated with the higher levels of C1QBP overexpression and perhaps a post-translationally modified form of C1QBP. The cell fractionation assay performed in HepG2 cells transfected with FLAG-tagged C1QBP or dMTS showed consistent results (S1B Fig). These findings indicate that C1QBP overexpression causes accumulation of immature C1QBP form in cells due to incomplete C1QBP mitochondrial translocation and MTS cleavage, while the observed higher molecular weight C1QBP warrants further characterization.

### Mapping the interaction domains between p22 and C1QBP

To determine the protein regions responsible for the p22-C1QBP interaction, various deletion mutants of C1QBP were tested in the co-IP assay, as illustrated in Fig 2D. The results demonstrated that the presence of aa 74–160 region of C1QBP supported C1QBP to interact with p22 (Figs 2E and S2A), and deletion of aa 74–160 completely abolished p22-C1QBP binding (Fig 2F). It is noteworthy that all three forms of overexpressed C1QBP-FLAG were capable of binding to p22 (Fig 2F, lane 2). Additionally, the dMTS mutant of C1QBP maintained interaction with p22 (S2B Fig), indicating that the MTS sequence or mitochondrial localization of C1QBP is not required for p22-C1QBP interaction.

**Fig 3:**
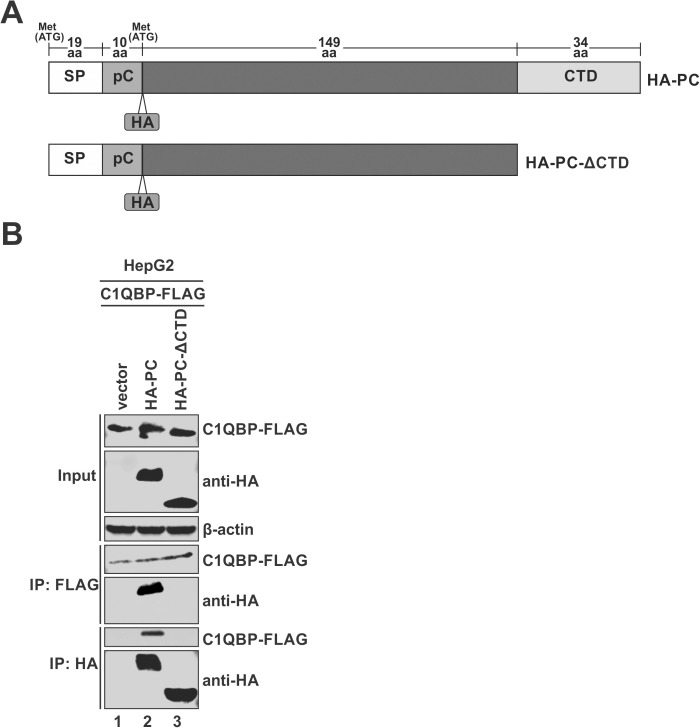
C1QBP-p22 interaction depends on the C-terminal domain (CTD) of p22. (A) Schematic representation of the full-length HA-PC and CTD truncation mutant HA-PC-ΔCTD. SP: signal peptide; pC: precore domain. (B) HepG2 cells were co-transfected with C1QBP-FLAG and either a control vector, HA-PC, or HA-PC-ΔCTD, for 3 days. 10% of cells were collected and lysed as input samples, while the remaining cells were subjected to co-IP using anti-FLAG or anti-HA antibody. The input and co-IP samples were subsequently analyzed by Western blot. β-actin was used as a loading control for input samples.

A previous study indicated that a truncated p22 lacking CTD failed to bind to C1QBP in HEK293 cells [35]. To further define the C1QBP-interacting domain on p22 in hepatocyte-derived cells, we tested a CTD-deletion mutant of HA-p22 in HepG2 cells co-transfected with C1QBP-FLAG (Fig 3A). Co-IP analysis demonstrated that the deletion of CTD completely abolished the binding of p22 with C1QBP, confirming the necessity of the CTD for this interaction (Fig 3B).

Given the arginine-rich, positively charged nature of the CTD of p22 and the distinct asymmetric electrostatic distribution of C1QBP homotrimer, we carried out AlphaFold3 modeling using the trimeric C1QBP in combination with either full-length p22 or its CTD to predict the interaction between C1QBP and p22. As shown in S3 Fig, the model with full-length p22 yielded a predicted template modeling (ipTM) value of 0.62 while the model using p22 CTD achieved a higher ipTM of 0.8, indicating a more confident, high-quality prediction. Both models revealed that the C-terminal region of CTD adopted a dipper-like fold, clustering several arginine residues to form a highly positively charged surface. This positively charged dipper inserts into the central cavity of the donut-shaped, negatively charged C1QBP, engaging with the inner wall formed by aa 74–160 (S3 Fig). The AlphaFold3 structural predictions are consistent with the above C1QBP-p22 interaction domain mapping results (Figs 2, 3 and S2).

### Assessing the subcellular localization of C1QBP-p22 interaction

**Fig 4:**
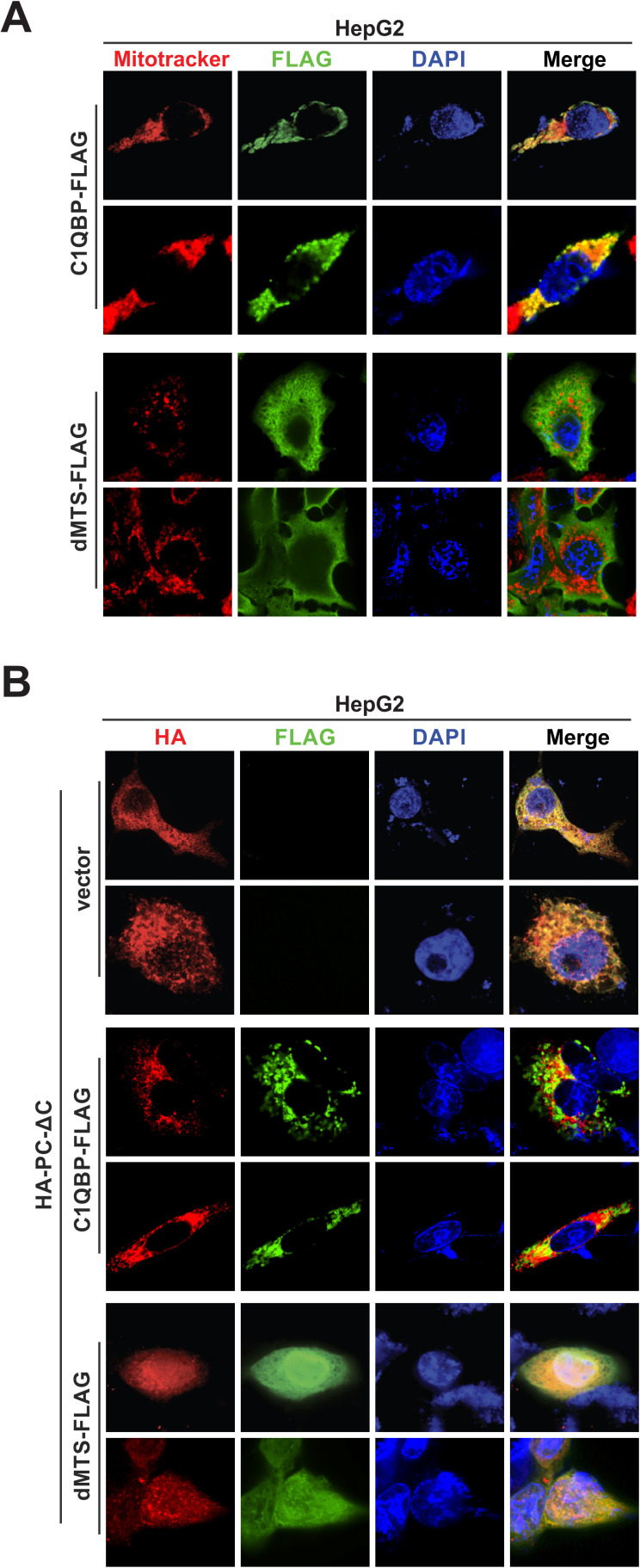
Subcellular localization of C1QBP-p22 interaction. (A) HepG2 cells were transfected with full-length C1QBP-FLAG or dMTS-FLAG for 48 h, followed by immunofluorescence analysis using anti-FLAG antibody (green). Mitochondria and nuclei were counterstained with MitoTracker (red) and DAPI (blue), respectively. Colocalizations were indicated by the yellow signals in the merged microscopy fields. (B) HepG2 cells were co-transfected with HA-PC-ΔC and control vector, C1QBP-FLAG, or dMTS-FLAG for 48 h, followed by immunofluorescence analysis using anti-HA (red) and anti-FLAG (green) antibodies. Cell nuclei were counterstained with DAPI (blue).

Consistent with the cell fractionation results (S1A Fig), immunofluorescence assay further revealed that the endogenous C1QBP is predominantly localized to the cytoplasm and mitochondria across various tissue-derived cell lines, including hepatoma cell line HepG2, lung adenocarcinoma cell line A549, and embryonic kidney cell line HEK293T, but also abundantly distributed in the nucleus of HEK293T cell (S4 Fig). Furthermore, the immunofluorescence analysis of C1QBP-FLAG and dMTS-FLAG in HepG2 cells showed that C1QBP-FLAG strongly colocalized with mitochondria (Fig 4A, upper panels), whereas dMTS-FLAG mutant exhibited a much-diffused cytoplasmic localization without clear co-localization with mitochondria (Fig 4A, lower panels). In agreement with these findings, the cytosol/mitochondria fractionation assay also demonstrated that dMTS-FLAG was not detected in the mitochondrial fraction (S1B Fig).

To define the specific cellular compartment in which the interaction between C1QBP and p22 occurs, HepG2 cells were co-transfected with HA-PC-ΔC (HA-PC with the ATG start codon of core ORF mutated to GTG to prevent HBc co-expression) and either a control vector, C1QBP-FLAG, or dMTS-FLAG, followed by immunofluorescence assay. The results demonstrated that p22 colocalized with both wt C1QBP and dMTS in the cytoplasm (Fig 4B). Interestingly, p22 was predominantly cytoplasmic in cells co-transfected with control vector or wt C1QBP (Fig 4B, upper and middle panels) but displayed a strong diffuse nuclear co-localization with dMTS in co-transfected cells (Fig 4B, lower panels). While dMTS alone did not show clear nuclear localization either (Fig 4A, lower panels), the enhanced p22-dMTS colocalization in the nucleus indicated that the p22-dMTS complex, once formed, may result in a synergistic effect on their nuclear import (Fig 4B, lower panels), and that the p22-C1QBP interaction may be multicompartmental and multifunctional in the cell.

### C1QBP inhibits HBV nucleocapsid formation in a p22-dependent manner

**Fig 5:**
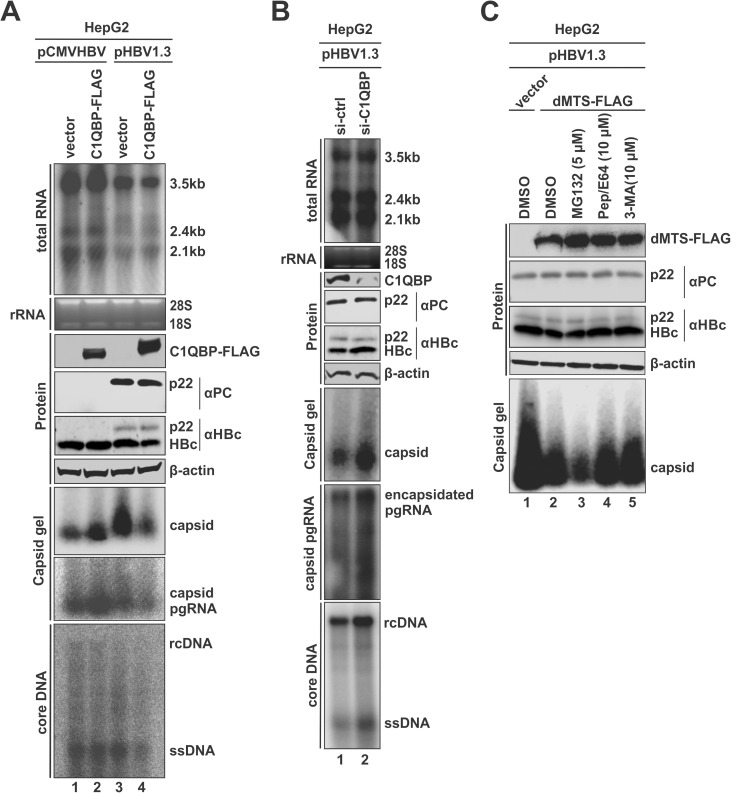
C1QBP inhibits HBV replication primarily through promoting autolysosomal degradation of HBc in a p22-dependent manner. (A) HepG2 cells were co-transfected with pCMVHBV or pHBV1.3 together with a control vector or C1QBP-FLAG. Cells were harvested on day 5 post-transfection. Intracellular HBV total RNA (3.5kb, 2.4kb, and 2.1kb) was analyzed by Northern blot. Ribosomal RNA (rRNA, 28S and 18S) was stained by ethidium bromide and served as a total RNA loading control. C1QBP-FLAG, HBc, and p22 levels were detected by Western blot, with β-actin serving as the loading control. The cytoplasmic HBV total capsids and their pgRNA content were analyzed by capsid gel assay. Cytoplasmic HBV core DNA was extracted and analyzed by Southern blot. (B) HepG2 cells were transfected with control siRNA (si-ctrl) or C1QBP siRNA (si-C1QBP) for 24 h, followed by pHBV1.3 transfection for an additional 5 days. HBV total RNA and encapsidated pgRNA were analyzed by Northern blot. C1QBP knockdown efficiency, HBc, p22, and β-actin levels were assessed by Western blot. Cytoplasmic HBV capsids were detected by capsid gel assay. HBV core DNA was analyzed by Southern blot. (C) HepG2 cells were co-transfected with pHBV1.3 and control vector or dMTS-FLAG. Two days after transfection, cells were treated with solvent DMSO, 5 μM of proteasome inhibitor MG132, 10 μM of lysosome inhibitor Pep/E64, or 10 μM of autophagy inhibitor 3-MA, as indicated, for 16 h. The levels of dMTS-FLAG, HBc, p22, and β-actin were analyzed by Western blot. The cytoplasmic HBV capsid levels were assessed by capsid gel assay.

Next, we examined the potential effect of cytosolic p22-C1QBP interaction on HBV replication. HepG2 cells were transfected with either the replication-competent HBV plasmid pHBV1.3 expressing all HBV proteins or pCMVHBV without p22 expression due to the lack of precore mRNA transcription [40–43], along with control or C1QBP-FLAG expression vector. As shown in Fig 5A, C1QBP overexpression did not affect the HBV replication derived from the pCMVHBV replicon (lanes 1–2). In contrast, it markedly inhibited HBV core DNA replication derived from the pHBV1.3 replicon (bottom panel, lane 4 vs 3). When the pHBV1.3-derived HBV replication steps upstream of reverse transcription were analyzed, the results demonstrate that C1QBP overexpression proportionally reduced the levels of encapsidated pgRNA, capsid, and HBc protein without significantly affecting viral RNA levels (upper panels, lane 4 vs 3). As expected, p22 was only detected under pHBV1.3 transfection, but interestingly, p22 expression was not affected by C1QBP overexpression, indicating a specific effect of C1QBP on HBc in the presence of p22 (middle panel, lane 4 vs 3). Furthermore, in the presence of lamivudine (3TC), which inhibits HBV reverse transcription in viral nucleocapsid, C1QBP overexpression still reduced the levels of HBc, capsid, and encapsidated pgRNA in pHBV1.3-transfected cells without affecting HBV total RNA level (S5A Fig). These results revealed a primary antiviral effect of C1QBP on HBc or nucleocapsid assembly in the context of pHBV1.3 transfection, and the differential inhibitory effect of C1QBP between pCMVHBV and pHBV1.3 indicates a p22-dependent mechanism.

To further assess the impact of endogenous C1QBP on HBV replication, we performed siRNA knockdown of C1QBP in pHBV1.3-transfected HepG2 cells. Silencing of C1QBP markedly enhanced the level of HBc protein and subsequent capsid formation, pgRNA encapsidation, and core DNA synthesis, while the levels of total viral RNA and p22 remained unchanged (Fig 5B). These findings indicate that the endogenous C1QBP acts as a negative regulator of HBV replication through reducing HBc level in the presence of p22. To specifically assess the antiviral effect of cytosolic C1QBP, the dMTS mutant was employed. Compared to C1QBP-FLAG, dMTS-FLAG overexpression resulted in a more pronounced reduction of HBc protein and downstream replication events without impact on HBV total RNA level (S5B Fig), indicating that the cytosolic form of C1QBP acts as the antiviral factor through interacting with p22. Furthermore, luciferase reporter assay showed that overexpression of wt C1QBP or dMTS did not alter HBV core promoter activity, confirming that the observed antiviral effect of C1QBP is irrelevant to pgRNA transcription (S5C Fig).

To explore the mechanism underlying C1QBP/p22-mediated HBc reduction, we treated dMTS-overexpressing cells with proteasome inhibitor MG132, lysosome inhibitors Pepstatin A and E64, or autophagy inhibitor 3-MA. While all the above inhibitors slightly increased the levels of dMTS-FLAG, the lysosome or autophagy inhibitor, but not the proteasome inhibitor, restored HBc protein and capsid levels in pHBV1.3-transfected HepG2 cells (Fig 5C), indicating that the C1QBP/p22-mediated HBc/capsid degradation involves both autophagy and lysosome, likely the autolysosome pathway [44]. However, the levels of p22 remained unaffected across all conditions, indicating that p22 is stable amid the C1QBP-mediated HBc degradation. Collectively, the above results suggest that C1QBP targets HBc protein for autolysosomal degradation in a p22-dependent manner in the context of HBV replication, likely through the formation of unstable HBc/p22 polymers and chimeric capsid [45,46], leading to a reduction of HBV pgRNA encapsidation and reverse transcription.

### Disruption of HBV capsid assembly enables C1QBP-HBc interaction in the absence of p22

**Fig 6:**
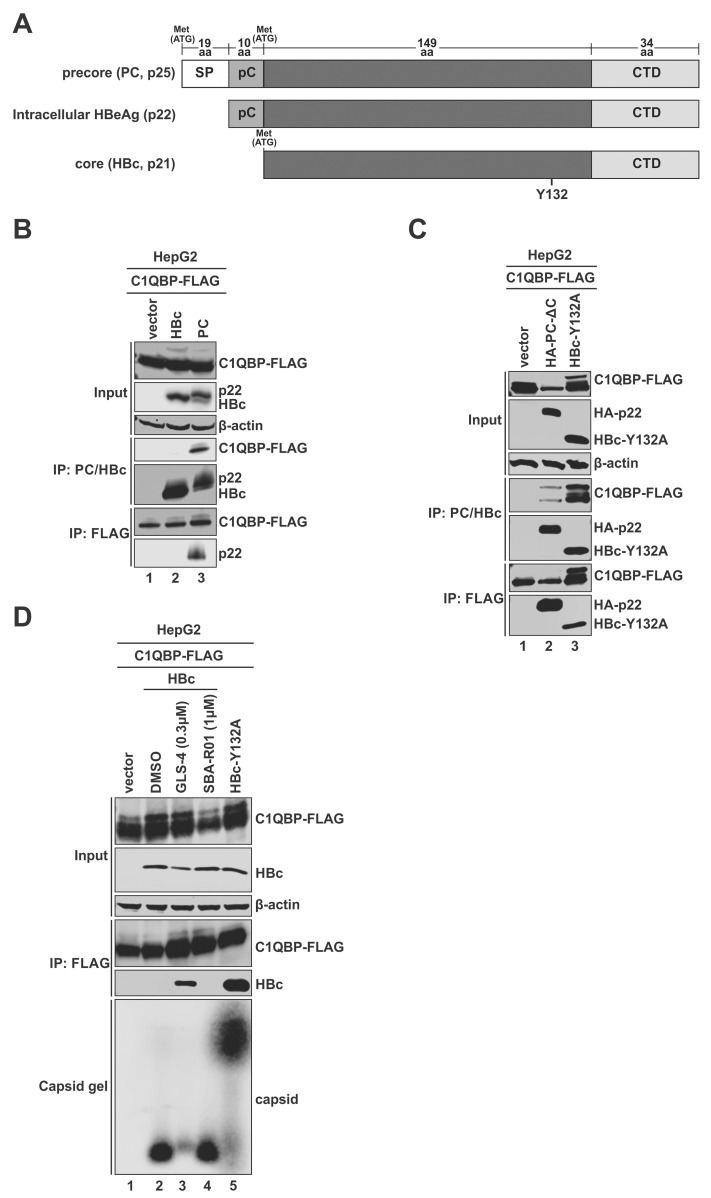
Disruption of HBV capsid assembly enables C1QBP-HBc interaction. (A) Schematic illustration of HBV PC protein (p25), p22, and HBc (p21). The position of Y132 on HBc is indicated. (B) HepG2 cells were co-transfected with C1QBP-FLAG and either a control vector, HBc, or PC for 3 days, followed by reciprocal co-IP assay with anti-HBc and anti-FLAG antibodies. The input and co-IP samples were analyzed by Western blot of HBc, p22, and C1QBP-FLAG. β-actin was used as a loading control for input samples. (C) HepG2 cells were co-transfected with C1QBP-FLAG plus a control vector, HA-PC-ΔC, or HBc-Y132A for 3 days, followed by reciprocal co-IP with anti-HBc and anti-FLAG antibodies. The input and co-IP samples were subjected to Western blot analysis of HBc, p22, and C1QBP-FLAG. β-actin served as a loading control for input samples. (D) HepG2 cells were co-transfected with C1QBP-FLAG and either a control vector, HBc, or HBc-Y132A. The C1QBP-FLAG/HBc-transfected cells were treated with solvent DMSO, 0.3 μM of GLS-4, or 1 μM of SBA-R01. Three days later, the cells were subjected to co-IP using anti-FLAG antibody. The input and co-IP samples were analyzed by Western blot using anti-HBc and anti-FLAG antibodies. β-actin served as a loading control for input samples. Cytoplasmic HBV capsid formation was assessed by capsid gel assay.

Given that HBc shares significant sequence similarity with precore and p22 proteins, especially the same CTD sequence responsible for p22-C1QBP interaction (Figs 3, S3 and 6A), we tested if HBc interacts with C1QBP. However, co-IP assay in HepG2 cells overexpressing C1QBP-FLAG and HBc showed no direct interaction, while p22 clearly bound C1QBP (Fig 6B). We reasoned that the failure of HBc-C1QBP interaction arises from the steric inaccessibility of the CTD region buried within the assembled capsid shell [47], while the CTD of p22 remains accessible for host protein binding [12]. In this regard, it has been reported that p22 interacts with HBc to form hybrid capsid [45,46], which may render C1QBP binding to the HBc/p22 complex. To test this hypothesis, we first confirmed the interaction between p22 and HBc using co-IP assay (S6A Fig). Moreover, we found that dMTS (the cytosolic form of C1QBP) interacted with HBc when p22 was co-expressed (S6B Fig), potentially through the formation of C1QBP-p22-HBc ternary complex. Interestingly, dMTS did not appear to induce HBc degradation in the presence of p22 but in the absence of HBV pgRNA and Pol, suggesting that, in addition to p22, other components of viral nucleocapsid are required for C1QBP-mediated HBc degradation.

Furthermore, we employed the HBc Y132A mutant that is known to impair capsid formation by disrupting dimer-dimer interactions critical for the formation of icosahedral capsid structures but to form abnormal HBc aggregates [48], which leaves the CTD being accessible. As shown in Fig 6C, co-IP assays showed that HBc-Y132A strongly interacted with C1QBP compared to wt HBc.

To further confirm the role of capsid integrity in restricting C1QBP-HBc interaction, we treated HBc-expressing cells with capsid assembly modulators (CAMs) possessing different mechanisms of action [49]. Treatment by GLS-4, a potent CAM-A compound inducing formation of aberrant HBc aggregates and subsequent degradation [50,51], resulted in a robust HBc-C1QBP interaction; whereas SBA-R01, a CAM-E compound that allows normal capsid assembly but blocks pgRNA encapsidation [52], failed to demonstrate HBc-C1QBP interaction (Fig 6D, lanes 1–4). Notably, HBc-Y132A formed aberrant protein aggregates/complexes rather than normal capsids (Fig 6D, bottom panel, lane 5).

The above findings demonstrated that the accessibility of CTD is essential for C1QBP binding, and that capsid disassembly or structural destabilization exposes the CTD, thereby facilitating the HBc-C1QBP interaction.

### C1QBP interacts with DP-rcDNA-containing capsid

**Fig 7:**
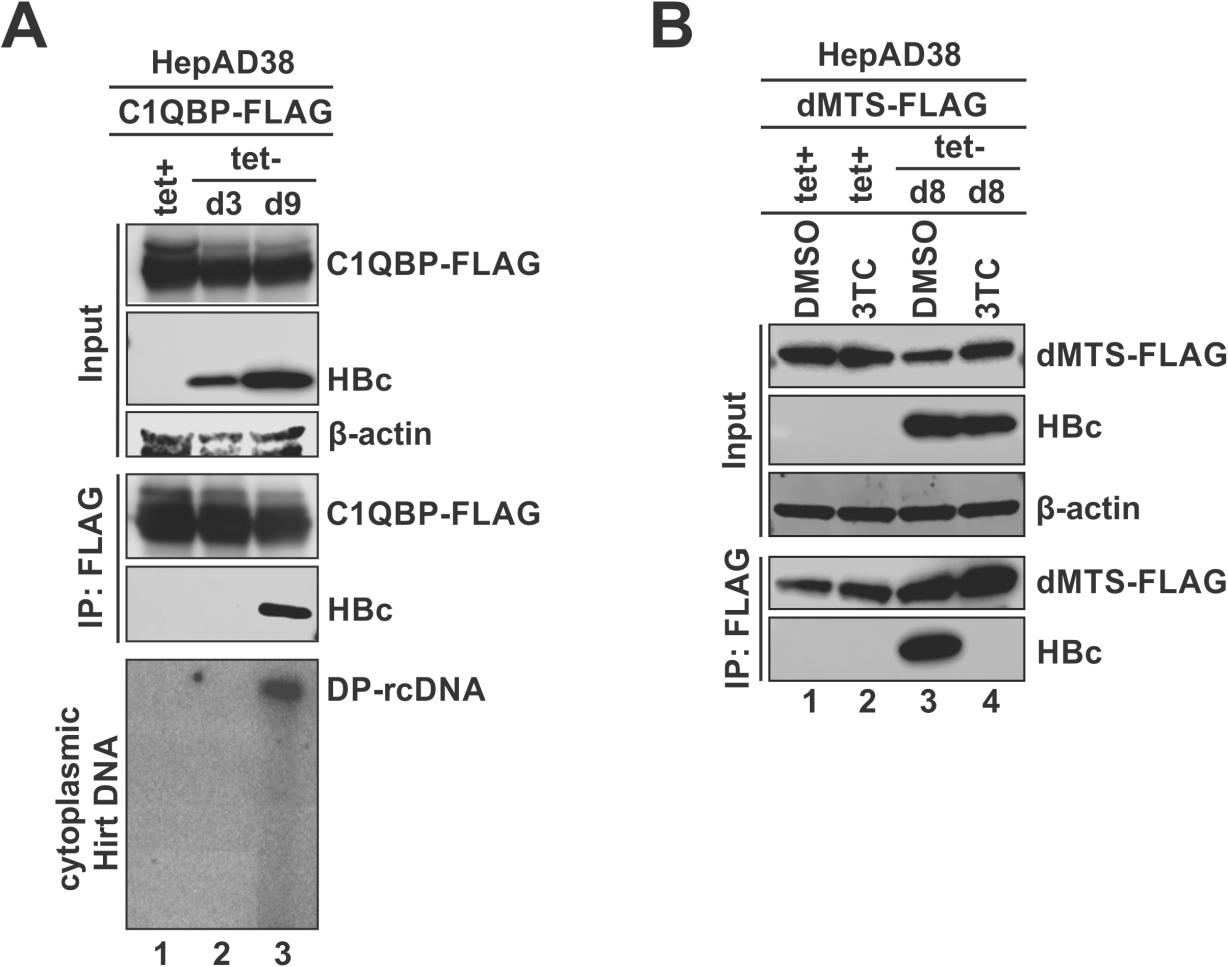
C1QBP interacts with DP-rcDNA-containing capsid. (A) HepAD38 cells were uninduced (tet+) or induced (tet-) for 0 or 6 days, followed by C1QBP-FLAG transfection for an additional 3 days in the presence or absence of tet as indicated. The harvested cells were subjected to co-IP using anti-FLAG antibody. The levels of C1QBP-FLAG and HBc in the input and co-IP samples were analyzed by Western blot. β-actin was used as a loading control for input samples. Cytoplasmic Hirt DNA was extracted and analyzed by Southern blot. (B) HepAD38 cells were treated with either DMSO or 10 μM of 3TC and maintained under uninduced (tet+) or induced (tet-) conditions for 0 or 5 days, as indicated. Subsequently, cells were transfected with dMTS-FLAG for an additional 3 days, maintaining the treatment as indicated. The harvested cells were analyzed by co-IP and Western blot as described in panel A.

Our previous study indicates that during HBV replication, around 90% of the cytoplasmic nucleocapsids containing deproteinized rcDNA (DP-rcDNA, also known as protein-free rcDNA [53]) undergo partial disassembly, resulting in the exposure of CTD and subsequent nuclear import of DP-rcDNA for cccDNA formation [54,55]. Given the above observation that C1QBP binds to HBc only when capsid assembly is disrupted (Fig 6C, D), we hypothesized that C1QBP may interact with DP-rcDNA-containing capsid. To test this hypothesis, HepAD38 cells were induced for 9 days to accumulate DP-rcDNA-containing capsids; the noninduced HepAD38 cells (HBc/nucleocapsid-negative) and cells induced for 3 days (HBc/nucleocapsid-positive, no detectable DP-rcDNA) served as controls. As expected, Southern blot assay showed that cytoplasmic DP-rcDNA was detected after 9 days of induction (tet-), but not in the noninduced cells (tet+) or cells induced for only 3 days (Fig 7A, bottom panel). Co-IP assay demonstrated that the transfected C1QBP-FLAG interacted with HBc only in the cells with detectable cytoplasmic DP-rcDNA (Fig 7A, upper and middle panels), indicating that the CTD exposure of HBc on DP-rcDNA-containing capsid enables HBc-C1QBP interaction. We further assessed C1QBP-HBc interaction in the induced HepAD38 cells under 3TC treatment. As shown in Fig 7B, the transfected dMTS-FLAG interacted with HBc only in the induced HepAD38 cells treated with DMSO solvent but not 3TC, suggesting that, by blocking HBV DNA replication and subsequent nucleocapsid disassembly, the HBc-C1QBP interaction cannot happen.

### C1QBP inhibits DP-rcDNA nuclear import and subsequent cccDNA formation

**Fig 8:**
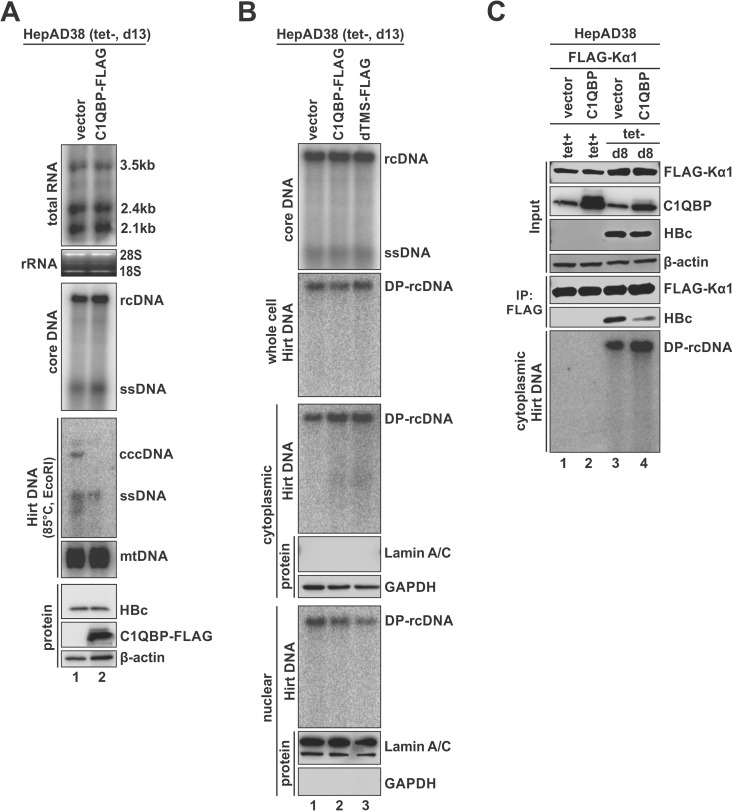
C1QBP inhibits nuclear import of HBV DP-rcDNA and subsequent cccDNA formation. (A) HepAD38 cells were induced for 10 days and then transfected with control vector or C1QBP-FLAG for an additional 3 days. Cells were subjected to HBV total RNA Northern blot and core DNA Southern blot analyses. Whole cell Hirt DNA was extracted and heat denatured at 85 °C for 10 min, EcoRI digested, and subjected to Southern blot analysis of HBV Hirt DNA and mitochondrial DNA (mtDNA). The indicated cccDNA and ssDNA represent the EcoRI-linearized cccDNA and heat-denatured DP-rcDNA, respectively. HBc and C1QBP-FLAG were detected by Western blot, with β-actin serving as the loading control. (B) HepAD38 cells were induced for 10 days and then transfected with either a control vector, C1QBP-FLAG, or dMTS-FLAG for an additional 3 days. The harvested cells were subjected to the following analyses: 1) Southern blot of cytoplasmic HBV core DNA; 2) Southern blot of whole-cell HBV Hirt DNA; and 3) cell fractionation to separate cytoplasmic and nuclear compartments. The purity of each fraction was confirmed by Western blot using the cytoplasmic marker GAPDH and the nuclear marker Lamin A/C. HBV Hirt DNA was then extracted from each fraction and analyzed by Southern blot. (C) HepAD38 cells were either uninduced (tet+) or induced (tet-) for 6 days and then co-transfected with FLAG-tagged karyopherin α1 (FLAG-Kα1) and a control vector or C1QBP for an additional 2 days. The harvested cells were subjected to co-IP using anti-FLAG antibody, and the input and co-IP samples were analyzed by Western blot using anti-FLAG, anti-C1QBP, and anti-HBc antibodies as indicated. β-actin served as the loading control for input samples. HBV cytoplasmic Hirt DNA was analyzed by Southern blot.

Exposure of the NLS-containing CTD of HBc is required for nuclear import of HBV capsid and its DNA contents [55–57]. To assess whether HBc-C1QBP interaction affects the nuclear transport of DP-rcDNA and subsequent cccDNA formation, we overexpressed C1QBP in the induced HepAD38 cells. Our results revealed that C1QBP overexpression significantly reduced cccDNA production without reducing HBV RNA transcription or DNA replication, indicating that C1QBP might inhibit cccDNA formation via targeting DP-rcDNA-containing capsid (Fig 8A). The lack of inhibitory effect of C1QBP overexpression on HBV DNA replication under this experimental condition was due to the low to undetectable level of cccDNA-dependent p22 expression in HepAD38 cells [58] (Fig 8A, lower panel), a similar situation as pCMVHBV transfection (Fig 5A). Furthermore, cell fractionation analysis demonstrated that the overexpression of either full-length C1QBP or dMTS mutant led to the accumulation of cytoplasmic DP-rcDNA and reduction of nuclear DP-rcDNA without affecting the overall HBV core DNA or total intracellular DP-rcDNA levels (Fig 8B), suggesting that C1QBP interacts with DP-rcDNA-containing capsid and impedes the nuclear import of DP-rcDNA.

We previously reported that cellular karyopherins, including karyopherin α1 (Kα1), Kα2, and Kβ1, bind to cytoplasmic DP-rcDNA-containing capsid and direct its nuclear transportation for cccDNA formation [55]. Given the above observation that C1QBP inhibits the nuclear import of DP-rcDNA, we hypothesized that this inhibitory effect results from competitive binding of C1QBP and karyopherins to DP-rcDNA-containing capsid. To test this hypothesis, we overexpressed FLAG-Kα1 alone or together with C1QBP in HepAD38 cells induced for 8 days. The co-IP results demonstrated that C1QBP overexpression markedly reduced the association of HBc with FLAG-Kα1, which was accompanied by increased cytoplasmic DP-rcDNA accumulation (Fig 8C), indicating that C1QBP competitively interferes with Kα1 binding to HBc on the DP-rcDNA-containing capsid and the subsequent nuclear transportation.

### C1QBP overexpression suppresses cccDNA formation during HBV infection

**Fig 9:**
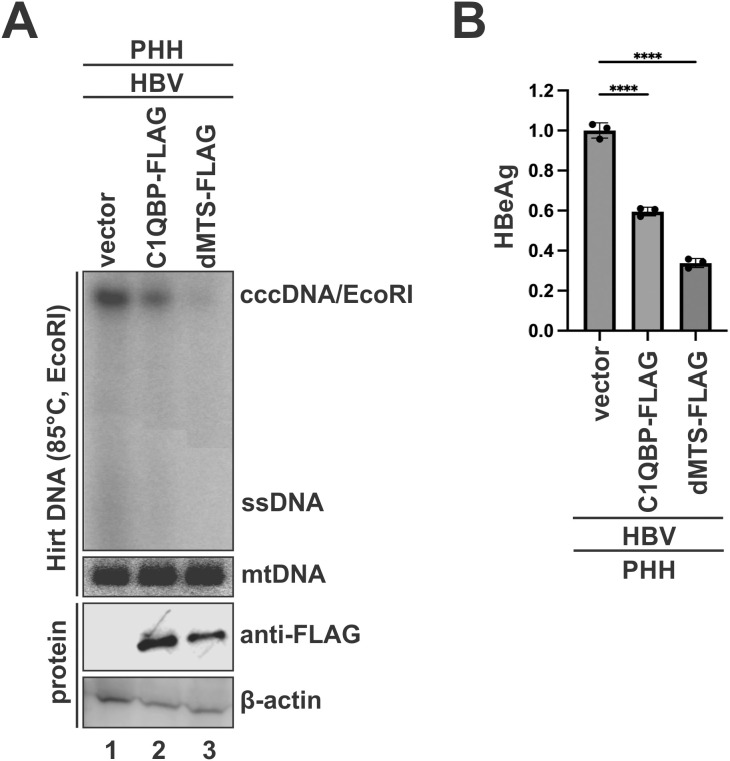
C1QBP overexpression inhibits cccDNA formation in HBV-infected PHHs. PHH cells were infected with HBV (100 MOI) for 24 h and then transfected with either a control vector, C1QBP-FLAG, or dMTS-FLAG, for an additional 7 days. (A) HBV Hirt DNA was extracted and detected by Southern blot. Before gel loading, Hirt DNA samples were denatured at 85 °C for 10 min, followed by EcoRI digestion. mtDNA was detected as loading control. Expression of C1QBP-FLAG and dMTS-FLAG proteins was confirmed by Western blot using anti-FLAG antibody, with β-actin serving as a loading control. (B) The levels of secreted HBeAg in cell culture supernatant were measured using chemiluminescent immunoassay (CLIA) and plotted as fold changes of the vector control group (mean ± SD, n = 3; ****p < 0.0001).

In HepAD38 cells, HBV cccDNA is formed exclusively through the rcDNA recycling pathway. To assess the potential effect of C1QBP on *de novo* cccDNA formation during HBV infection, C1QBP-FLAG or dMTS-FLAG was overexpressed in HBV-infected primary human hepatocytes (PHHs) via centrifugal transfection. The results demonstrated that C1QBP-FLAG overexpression significantly reduced cccDNA level and subsequent HBeAg production compared to the control, and consistently, dMTS-FLAG exhibited a more pronounced inhibitory effect (Fig 9), suggesting that C1QBP restricts HBV infection through impairing cccDNA formation.

## Discussion

HBV p22 is an intermediate protein involved in the biogenesis of HBeAg [10,11]. Following co-translational cleavage of the N-terminal signal peptide from the nascent precore (p25) protein, the resulting p22 enters the ER lumen and is subsequently transported to the Golgi complex, where furin-mediated cleavage of its CTD facilitates the secretion of HBeAg [11]. A large portion of p22 (approximately 50–80%) fails to translocate into the ER and remains in the cytosol [10,59]. Although cytosolic p22 has been reported to result from retro-translocation from the ER lumen via the ER-associated degradation (ERAD) pathway [60], the detailed mechanisms governing p22 biogenesis and distribution between ER and cytosol remain to be elucidated.

Cytosolic p22 is a nonstructural viral protein and dispensable for viral replication but can form defective heterocapsid through interacting with HBc in the cytoplasm, suggesting its potential role as a self-limiting viral protein in the HBV life cycle [45,46,61]. On the other hand, accumulating evidence suggests that the cytosolic p22 engages in a broad network of interactions with host proteins. It has been reported that the cytosolic p22 interacts with Toll/IL-1 receptor (TIR)-containing proteins TRAM, Mal, and TLR2 to suppress TLR activation [62]. Our previous study demonstrated that p22 competitively binds karyopherin α1, thereby blocking nuclear translocation of STAT1/2 and subsequently suppressing JAK/STAT signaling [43]. Additionally, p22 has been reported to interact with NUMB, thereby inhibiting p53 activity [63]. Based on these findings, HBV may exploit the p22-mediated suppression of host innate immunity, cell proliferation, and viral replication to establish persistent infection and shape the pathogenesis of chronic hepatitis B. This notion aligns well with clinical observations, wherein the HBV precore G1896A stop codon mutation, characterized by precore/HBeAg-negative, is frequently associated with more severe liver disease manifestations [64–66].

In this study, by using the HepHA-HBe4 cells constitutively expressing HA-tagged intracellular p22 [36], we conducted an anti-HA pull-down assay coupled by LC-MS analysis. For the first time, we obtained a preliminary p22 interactome and re-identified C1QBP as a p22-interacting protein (Figs 1–4). Beyond proteins associated with ribosomal function and cytoskeletal organization, the identified POIs include signaling and regulatory proteins such as AMOT (Angiomotin), BCAR1 (Breast Cancer Anti-Estrogen Resistance Protein 1), Calmodulin, and PARP1 (Poly [ADP-ribose] polymerase 1), implying a potential role of p22 in modulating host signaling pathways. Moreover, interactions between p22 and host nuclear proteins, such as NCL (Nucleolin), PARP1, and ERH (Enhancer of rudimentary homolog), indicate a possible involvement of p22 in regulating host and/or HBV DNA maintenance and transcription. It is conceivable that the assay did not score the previously reported p22-interacting STAT1/2 and TIR-containing proteins due to the absence of corresponding innate stimulations; however, NUMB was not reidentified by our assay. Finally, C1QBP was prioritized for further study based on its known interactions with HBV p22 and established roles in regulating several other viruses [28,37–39].

The full-length C1QBP protein consists of 282 aa (~32 kDa), and the first 73 aa encodes a mitochondrial targeting signal (MTS) that directs the protein to mitochondria [19,67]. In high frequency, the MTS is subsequently cleaved off to generate the mature form of C1QBP, which forms a donut-shaped homotrimer predominantly located in the mitochondrial matrix, but the leaked form can also be found in other subcellular compartments (S4 Fig) [20–23]. In our study, the distribution of endogenous C1QBP between mitochondria and cytosol was also observed in hepatocyte-derived cells using the subcellular fractionation assay, and overexpression of C1QBP led to the accumulation of immature forms presumably due to an inefficient and/or incomplete MTS cleavage (S1 Fig). In addition, the MTS-dependent mitochondrial localization of C1QBP was confirmed by both subcellular fractionation and immunofluorescence colocalization assays (Figs S1B and 4A).

The interacting domains between C1QBP and p22 were mapped to the N-terminal domain (aa 74–160) of mature C1QBP and the CTD of p22 (Figs 2, 3 and S2). C1QBP is a highly acidic protein, and its donut-shaped homotrimer forms a central pore approximately 20 Å in diameter, partially covered by flexible loop regions [20]. Notably, the trimer exhibits an asymmetrical electrostatic surface potential, characterized by a negatively charged face. This distinctive structure and polarized charge distribution likely underpin its extensive capacity for interacting with diverse proteins, including various viral and host factors [20]. Consistently, AlphaFold3 prediction suggests that the flexible, arginine-rich, positively charged CTD of p22 interacts with the negatively charged central channel (aa 74–160) of C1QBP, likely through salt bridges and other electrostatic interactions (S3 Fig). Future cryo-electron microscopy (cryo-EM) studies are needed to validate these structural predictions of p22-C1QBP interaction. Moreover, whether this structural model also applies to the reported interactions between C1QBP and other viral proteins remains to be further investigated. It is also possible that additional host proteins may serve as co-factors to facilitate the interaction between C1QBP and viral proteins.

C1QBP has been reported to interact with several herpesviral proteins intracellularly, playing diverse roles in viral life cycles. For example, C1QBP association with IE63 of HSV-1 could interfere with pre-mRNA splicing, aiding nuclear export of viral transcripts [68]. Additionally, C1QBP binds ICP34.5 of HSV-1 to form a complex with PKCδ, subsequently promoting the nuclear egress of viral capsids [32]. In EBV infection, C1QBP interacts with EBNA-1 and localizes to the viral replication origin (oriP), contributing to EBNA-1-mediated transcription activation [29,37]. For CMV, C1QBP binds the viral pUL97 protein, enhancing nuclear capsid export [30]. Interestingly, while these herpes viruses appear to exploit C1QBP to enhance their replication, our study revealed an inhibitory effect of cytosolic C1QBP on HBV nucleocapsid formation (Figs 5 and S5). Such an antiviral effect is p22-dependent and thus likely mediated by the interaction between p22 and HBc. In this regard, naturally occurring viral mutants that reduce or abolish p22/HBeAg production, such as the basal core promoter double mutation A1762T/G1764A and precore stop codon mutation G1896A [69], are expected to weaken the inhibitory effect of C1QBP. This speculation is consistent with the clinical observations that these mutations are frequently associated with higher viral loads and more severe disease progression.

Previous studies have shown that the dimer-dimer interaction between p22 and HBc resulted in an unstable chimeric capsid-like structure devoid of encapsidated pgRNA [12,45,46]. Therefore, the p22-interacting C1QBP may either facilitate or interrupt the p22-HBc chimeric capsid formation, but likely through the latter possibility, as C1QBP overexpression reduced the level of capsid and C1QBP siRNA knockdown exhibited the opposite effect (Fig 5A, B). It has been reported that the cytosolic p22 was co-degraded with HBc partially through proteasomal degradation [46]. We also found that C1QBP reduced HBc in a p22-dependent manner (Figs 5A, B and S5). However, the C1QBP-p22-induced HBc degradation mainly involves autophagy and lysosome but not proteasome (Fig 5C), and p22 does not undergo co-degradation with HBc (Fig 5A–C). Notably, when dMTS, p22, and HBc were co-expressed in the absence of HBV replication, dMTS did not reduce the levels of HBc (S6B Fig). These findings indicate that a C1QBP-mediated HBc degradation mechanism exists in the presence of p22 and viral replication complex, whereby the C1QBP-p22 complex prevents HBc from forming normal nucleocapsid and causes lysosomal degradation of HBc; however, the p22-C1QBP complex is not subjected to the same HBc degradation route. Considering the C1QBP-p22 complex can bind to HBc and prevent nucleocapsid formation (Figs 5 and S6B), the increased unassembled HBc are targeted for autolysosomal degradation. In contrast, the C1QBP-p22-HBc ternary complex itself may be relatively stable and thus not subject to degradation, which could explain why C1QBP expression does not alter the overall p22 level. Another possibility is that the autolysosomal degradation apparatus may be able to extract HBc from the C1QBP-p22-HBc complex for degradation. A structural interpretation of this complex and its dynamics within the native cellular environment, utilizing *in situ* cryo-electron tomography (cryo-ET) coupled with cryo-focused ion beam (cryo-FIB) milling [70], is warranted.

The differential involvement of autolysosome and proteasome in p22- and p22-C1QBP-mediated HBc degradation between our study and the previous study [46], respectively, could be due to the different stoichiometry between the three proteins or experimental conditions. For example, our experiment was conducted in the context of pHBV1.3 transfection, and the previous study was done under p22 and HBc co-transfection [46]. Moreover, HBc is generally thought to be resistant to the ubiquitination-dependent proteasomal degradation due to its low lysine content and the formation of stable icosahedral capsid [71,72], and autophagy has been implicated in the regulation of HBV nucleocapsid assembly and precore protein activities [73,74]. In line with these, a previous study demonstrated that the aberrant HBc polymers formed under treatment of CAM-A compound Bay41–4109 is predominantly degraded by macroautophagy and lysosome [75].

HBc is the building block of HBV capsid; the monomeric HBc consists of a 140 aa N-terminal assembly domain, a short 9 aa linker, and a 34 aa arginine-rich CTD [76]. Although HBc and p22 share the same CTD sequence involved in p22-C1QBP interaction (Fig 6A), co-IP assay indicated no direct interaction between core protein and C1QBP in the absence of p22 (Fig 6B). This finding aligns with a previous report showing that NUMB, another p22-binding factor, does not interact with HBc despite sharing the direct binding domain (aa 150–165) [63]. The lack of interaction between either C1QBP or NUMB with HBc might result from significant steric hindrance imposed by the assembled capsid structure. Specifically, the CTD of HBc becomes structurally constrained within mature capsids, significantly limiting its accessibility by external factors [47]. To examine if capsid assembly affects CTD accessibility by C1QBP, we employed two strategies to disrupt normal capsid formation: 1) expression of the assembly-defective HBc mutant Y132A [48]; and 2) treatment with CAM-A compound GLS-4 to induce formation of aberrant HBc polymers [49]. The results showed that disruption of the normal capsid assembly by either introducing Y132A mutation or GLS-4 treatment, but not the normal empty capsid assembly facilitator CAM-E compound SBA-R01 [52], enabled the interaction between C1QBP and HBc, likely by exposing the CTD region typically sequestered within intact capsids [47] (Fig 6C–D). On the other hand, our experimental approaches using the HBc-Y132A mutant or CAM-A treatment could also be applicable for identifying additional host-binding factors interacting with the CTD of HBc.

In HBV life cycle, the disassembly of mature nucleocapsid (uncoating) is a mandatory step to release the rcDNA genome for cccDNA formation [77,78]. Our previous studies suggested that the cytoplasmic rcDNA deproteination could induce a partial disassembly of the nucleocapsid, leading to the exposure of HBc CTD for subsequent karyopherins-mediated nuclear import of DP-rcDNA containing capsid [55,79]. In this natural scenario, we also observed the interaction of C1QBP with HBc upon rcDNA deproteination in induced HepAD38 cells (Fig 7). The consequence of such interaction is that the cytosolic C1QBP competes with karyopherins for binding to the NLS-containing CTD of HBc on DP-rcDNA-containing capsid, thus hindering DP-rcDNA nuclear import and subsequent cccDNA formation (Fig 8). Additionally, C1QBP is able to inhibit cccDNA formation in HBV-infected PHH cells (Fig 9). Given that the recycling pathway for cccDNA formation is inefficient in *in vitro* HBV infection systems [80–82], the observed antiviral effect of C1QBP on cccDNA production in both HBV stable cell line HepAD38 and infection system suggests that C1QBP inhibits both *de novo* synthesis and intracellular amplification of cccDNA.

The plasma membrane-associated C1QBP is known to bind to human complement subcomponent C1q molecules and inhibit C1 activation [23]. This immunomodulatory function of C1QBP was not examined in this study due to the absence of complement system in HBV cell culture models. Whether C1QBP plays any role in host adaptive immune responses to HBV infection has not been studied so far. Although circulating HBeAg can impact host adaptive immunity *in vivo* [66,83,84], it cannot interact with C1QBP directly due to the loss of CTD (Fig 3). Moreover, a previous study has ruled out the involvement of C1QBP in HBeAg-mediated inhibition of T cell proliferation [85]. On the other hand, C1QBP has been shown to modulate cellular innate immunity during viral infections. For instance, C1QBP inhibits viral dsRNA-mediated activation of RIG-I and MDA5 by interacting with the adaptor protein MAVS on mitochondria, suppressing the downstream IRF3 and NF-κB signaling and type I IFN production [27]. Additionally, the cytosolic C1QBP interacts with the NTase domain of cGAS, inhibiting its enzymatic activity, and thereby suppressing the production of type I IFN, promoting HSV-1 infection [28]. However, in terms of HBV, the virus is generally considered as a stealth virus without being detected by the innate sensors or inducing IFN in hepatocytes [86–88]. Furthermore, there is a lack of functional innate-DNA sensing pathways in hepatocytes [87,89]. Hence, the observed antiviral effect of C1QBP on HBV replication in our study is unlikely to involve the reported innate immune suppressive activity of C1QBP.

Beyond the anti-HBV activity of cytosolic C1QBP, the potential effects of C1QBP-p22 interaction on other host functions remain to be investigated. HBV has been reported to target the mitochondria and alter mitochondrial integrity, metabolism, and function via multiple viral components and mechanisms [90–92]. Given the primary mitochondrial localization of C1QBP, it is of interest to examine the potential effect of p22 on mitochondria via p22-C1QBP interaction in future studies. In this regard, a recent study demonstrated that circulating HBeAg attenuates the antiviral response of M1-like macrophages via activating TLR4 to promote mitochondrial OXPHOS [93]. On the other hand, mitochondrial C1QBP acts as a critical factor to maintain OXPHOS [18,19]. Therefore, whether C1QBP plays a role in HBeAg-induced mitochondrial OXPHOS in macrophages, and whether the intracellular p22 alters mitochondrial function through interaction with C1QBP in hepatocytes, await further investigation.

**Fig 10:**
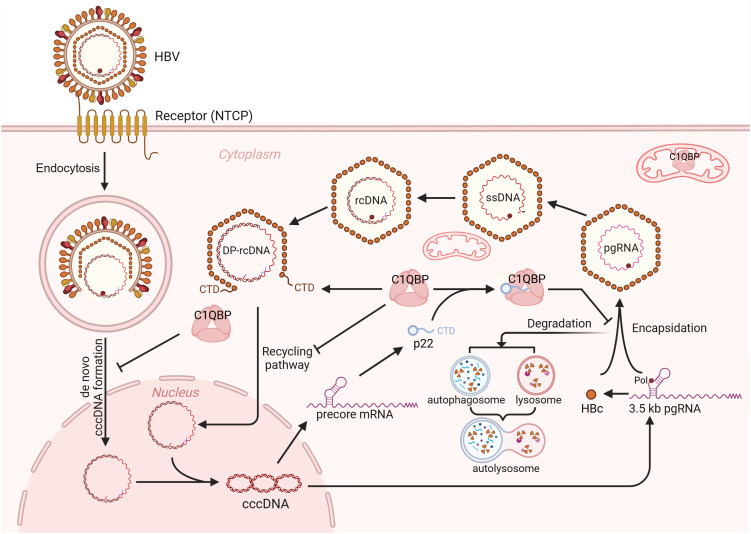
Mode of action of C1QBP-mediated inhibition of HBV replication. HBV life cycle is briefly illustrated, highlighting the key steps involved in viral replication. The 3.5kb precore mRNA and pgRNA transcribed from cccDNA serve as translational templates for p22 and HBc, respectively. The pgRNA encapsidation step, in which HBc assembles into nucleocapsids with pgRNA and Pol, is inhibited by the cytosolic C1QBP in a p22-dependent manner. Specifically, C1QBP-p22 complex binds to HBc and prevents it from assembling into nucleocapsid. The increased free unbound HBc proteins are targeted for autolysosomal degradation. Once encapsidated, the pgRNA undergoes reverse transcription to form ssDNA and then rcDNA. Partial disassembly of the capsid is triggered by deproteinization of rcDNA, resulting in the exposure of the CTD of HBc. The exposed CTD contains NLS that serves as a recognition site for nuclear import factors, including karyopherin α1 (Kα1), facilitating nuclear import of DP-rcDNA. During this process, C1QBP competitively interacts with the exposed CTD of HBc, thereby preventing Kα1 from binding to the capsid to impede DP-rcDNA nuclear import and subsequent cccDNA formation. See the text for more details. Created in BioRender (https://BioRender.com/hktl2f0).

In summary, our study identified two antiviral mechanisms of C1QBP against HBV infection (Fig 10). One is to induce the autolysosomal degradation of HBc in a p22-dependent manner, the other is to block nuclear import of DP-rcDNA-containing capsid and subsequent cccDNA formation in a HBc-dependent but p22-independent manner. This dual antiviral mechanism of C1QBP establishes it as a host restriction factor for HBV infection in both HBeAg-positive and -negative phases in the natural history of chronic hepatitis B, offering a potential target for future antiviral therapies aimed at curtailing viral persistence.

## Materials and methods

### Cell cultures

HepG2 and A549 cells were cultured in DMEM/F12 medium (HyClone, SH30023.01), HEK293T cells were maintained in DMEM medium (Corning, 10–013-CM), both were supplemented with 10% heat-inactivated fetal bovine serum (Atlanta Biologicals, S10350), 100 U/ml penicillin, and 100 μg/ml streptomycin. The tetracycline-inducible (tet-off) HBV stable cell lines HepAD38 [94], the HepHA-HBe4 stable cell line constitutively expressing HA-p22 and HA-HBeAg [36], and the HepG2-NTCP cells [95], were maintained as previously described. To induce HBV replication in HepAD38 cells, tet was removed from the culture medium and cells were cultured in tet-free medium for indicated time duration. Freshly isolated and plated primary human hepatocytes (PHHs) were provided by the Human Liver Tissue and Hepatocyte Research Resource (HLTHRR) at the University of Pittsburgh (supported by NIDDK project# 1R24DK139775–01) and cultured as described before [82]. All cell cultures were maintained at 37°C in an incubator with 5% CO_2_.

### Plasmids, siRNA, and transfection

HBV (genotype D) replicon plasmid pHBV1.3 containing a 1.3-mer viral genome and pCMVHBV transcribing HBV pgRNA under the control of a human cytomegalovirus immediate early (CMV-IE) promoter were described previously [40,41,96]. Expression plasmids for HBV precore/core proteins, including pcHBc that expresses core protein (HBc), pcHBe expressing precore/HBeAg proteins (PC), pcHA-HBe expressing HA-tagged precore/HBeAg (HA-PC), pcHA-HBe-ΔCTD expressing HA-PC without the CTD domain (HA-PC-ΔCTD), have been described in our previous publications [36,43]. Plasmid HBc-Y132A expressing a Y132A mutant of HBc was constructed from pcHBc by substituting tyrosine at aa 132 with alanine within the HBc ORF. Plasmid HA-PC-ΔC was derived from HA-PC with the start codon (ATG) of core ORF being substituted with GTG.

The ORF sequence of full-length C1QBP (GenBank Accession No: NM_001212.4) was cloned into the pcDNA3.1(+) vector to generate plasmid pcC1QBP. Plasmid expressing the MTS deletion mutant of C1QBP, pcC1QBP-dMTS (also known as dMTS), was generated by removing aa 2–73 from the C1QBP ORF on pcC1QBP. Plasmids encoding C-terminally FLAG-tagged full-length and truncated C1QBP proteins (C1QBP-FLAG, dMTS-FLAG, 1–160-FLAG, 1–220-FLAG, 74–160-FLAG, and 74–220-FLAG) were described previously [28]. Δ74–160-FLAG deletion mutant was constructed by deleting the p22-interaction domain (aa 74–160)-coding sequence from plasmid C1QBP-FLAG. Plasmid expressing N-terminally FLAG-tagged karyopherin α1 (FLAG-Kα1) was provided by Dr. Christopher Basler through BEI Resources [97].

The HBV enhancer II/core promoter firefly luciferase reporter plasmid EnII/Cp-Luc was described previously [98]. The CMV-IE promoter *Renilla* luciferase reporter plasmid pRL-CMV was purchased from Promega (E2261).

Plasmid transfection was performed with Lipofectamine 3000 transfection reagent (Invitrogen, L3000150). To enhance transfection efficiency in PHHs, the cell culture plates were centrifuged at 1,000 rpm for 15 min at room temperature (RT) immediately after the addition of the transfection mixture to the plated PHHs, followed by transferring the plates into the cell incubator. The centrifugal transfection efficiency was monitored by transfecting an enhanced green fluorescent protein (eGFP) expression plasmid (S7 Fig).

Control siRNA-A (sc-37007) and C1QBP siRNA (sc-42880) were purchased from Santa Cruz Biotechnology. siRNA transfection was carried out using Lipofectamine RNAiMAX Transfection Reagent (Thermo Fisher Scientific, 13778100) according to the manufacturer’s instructions.

### Compounds

HBV capsid assembly modulators (CAM) GLS-4 and SBA-R01 were provided by Arbutus Biopharma [51,99]. Lamivudine (3TC) was provided by Dr. William Mason (Fox Chase Cancer Center). Proteasome inhibitor MG132 (HY-13259), lysosomal inhibitors Pepstatin A (HY-P0018) and E64 (HY-15282), and autophagy inhibitor 3-Methyladenine (3-MA, HY-19312), were purchased from MedChemExpress.

### HBV infection

HBV particles were collected from the supernatant of induced HepAD38 cells, and the virion titer was determined according to our previous publication [95]. HBV infection of HepG2-NTCP and PHH cells was performed as previously described [43].

### HBV nucleic acids analysis

The cytoplasmic HBV capsid-associated DNA (core DNA), whole cell, cytoplasmic, and nuclear HBV Hirt DNA (DP-rcDNA and cccDNA) were isolated, prepared, and detected by Southern blot as previously described [54,55,100]. HBV total RNA and encapsidated pgRNA were isolated and subjected to Northern blot following our previous studies [101,102]. Hybridization signals were captured using a phosphorimager screen and scanned by the Typhoon FLA-7000 imaging system (GE Healthcare).

### HBV particle gel assay

The intracellular HBV capsids were detected by particle gel enzyme immunoassay (EIA) following our published protocol [103]. The *in-situ* detection of encapsidated pgRNA on particle gel membrane was conducted as described previously [101,102].

### HBeAg chemiluminescent immunoassay (CLIA)

HBeAg in the cell culture supernatant was measured using the HBeAg CLIA Kit (Ig Biotechnology, CL18004) according to the manufacturer’s instructions.

### Dual-luciferase reporter assay

Promoter activity was evaluated using the Dual-Luciferase Reporter Assay System (Promega, E1910) following the manufacturer’s protocol. Relative firefly luciferase reporter activities were normalized to *Renilla* luciferase signals.

### Subcellular fractionation

Mitochondria isolation was performed using Mitochondria Isolation Kit (Thermo Fisher Scientific, 89874) according to the manufacturer’s manual. NE-PER Nuclear and Cytoplasmic Extraction Reagents (Thermo Fisher Scientific, 78835) was employed to separate nuclear and cytoplasmic fractions.

### Western blotting

Whole cell lysate samples were prepared using Laemmli buffer and sonicated with an EpiShear ultrasound sonicator (Active Motif, 53051). The samples were denatured at 95°C for 10 min and separated in precast SDS-PAGE gels (Invitrogen), and transferred to nitrocellulose membranes (Cytiva, 10600002). The membranes were blocked with 5% non-fat dry milk (Research Products International, M17200-1000.0) for 1 h at RT and probed with corresponding primary antibodies anti-precore/core (Dako, B0586), anti-precore mAb 1A11 (provided by Dr. Peter Revill at Peter Doherty Institute for Infection and Immunity) [104,105], anti-C1QBP (Cell Signaling Technology, 6502), anti-HA (Cell Signaling Technology, 2367 or 3724), anti-FLAG (Millipore Sigma, F7425), anti-β-actin (Santa Cruz, sc-47778), anti-VDAC (Cell Signaling Technology, 4866), anti-GAPDH (Santa Cruz, sc-47724), and anti-Lamin A/C (Santa Cruz, sc-376248). After IRDye-conjugated secondary antibodies incubation, the signals were visualized using the Li-COR Odyssey imaging system.

### Co-immunoprecipitation (co-IP)

Cells from 10-cm plates were rinsed with 1 × PBS and harvested in 1 ml lysis buffer (1% NP-40, 10% glycerol, 2 mM EDTA, 50 mM Tris-HCl [pH 7.0], and 150 mM NaCl) supplemented with 1 × Halt protease inhibitor cocktail (Thermo Fisher Scientific, 87785) and 100 μM PMSF (Millipore Sigma, 52332). 10% of the cell lysates were used as input. The remaining cell lysates were incubated with rotation at 4°C for 1 h, and then centrifuged at 4°C for 10 min. The clarified lysates were then mixed with indicated antibodies and rotated at 4°C for 1 h. For each cell lysate, 20 μl of protein A/G beads (Santa Cruz, sc-2003) were washed three times with lysis buffer and added to the lysate-antibody mixture. For anti-FLAG immunoprecipitation, Anti-FLAG M2 Affinity Gel (Millipore Sigma, A2220) was used. For anti-HA immunoprecipitation, EZview Red Anti-HA Affinity Gel (Millipore Sigma, E6779) was used. After incubation at 4°C overnight, the beads were precipitated by centrifugation and washed three times with lysis buffer. After the final wash, the supernatant was discarded and 40 μl of lysis buffer was added, followed by the addition of 10 μl of 5 × SDS loading buffer (250 mM Tris-HCl [pH 6.8], 10% SDS, 50% glycerol, 500 mM DTT, and 0.5% Bromophenol Blue) for subsequent Western blot analysis.

### Immunofluorescence

Cells were seeded onto glass coverslips in a 6-well plate and allowed to adhere overnight. On the following day, the cells were fixed with 4% paraformaldehyde (Santa Cruz, sc-281692) for 10 min at RT, followed by three washes with PBS. After fixation, permeabilization was performed using 0.1% Triton X-100 in PBS for 10 min at RT. Then, the cells were incubated in blocking buffer (4% bovine serum albumin in 1 × PBS) for 1 h at RT. Primary antibodies, diluted in blocking buffer, were then applied at dilution of 1:200 in blocking buffer and incubated at 4°C overnight. After primary antibody incubation, cells were washed three times with PBS and incubated with goat anti-rabbit IgG (H + L) cross-adsorbed secondary antibody, Alexa Fluo 488 (Thermo Fisher Scientific, A11008) or goat anti-mouse IgG (H + L) cross-adsorbed secondary antibody, Alexa Fluor 594 (Thermo Fisher Scientific, A11005), diluted in blocking buffer, for 1 h at RT in the dark. Following secondary antibody incubation, the cells were washed three times with PBS and counterstained with DAPI (Thermo Fisher Scientific, 62248). To label mitochondria, cells were stained with MitoTracker Deep Red FM (Thermo Fisher Scientific, M22426) according to the manufacturer’s instructions. Coverslips were then mounted onto microscope slides and visualized using a confocal Olympus IX81 equipped with a spinning-disk confocal head using Olympus CellSense software.

### HBV p22 co-IP and LC-MS analysis

HepHA-HBe4 cells, which constitutively express HA-tagged p22/HBeAg [36], and control HepG2 cells were cultured and harvested separately. The co-IP was performed by using the Universal Magnetic Co-IP Kit (Active Motif, 54002). Briefly, cells were lysed using the protease inhibitor-containing lysis buffer provided in the kit. The clarified lysates were incubated with either anti-HA antibody and protein G magnetic beads or beads alone for 3 h at 4°C with constant and gentle agitation. Extensive washing was then carried out according to the manufacturer’s instructions. The beads-bound proteins were reduced, alkylated, and trypsin digested directly on beads, and the subsequent peptides were identified with liquid chromatography interfaced with a hybrid quadrupole-Orbitrap mass spectrometer (ThermoFisher Q Exactive HF Orbitrap LC-MS/MS system) at the Purdue University Proteomics Facility. Database search against the Uniprot human database was conducted using MaxQuant to identify the probable source proteins of the peptides. Proteins specifically identified from the anti-HA pull-down of HepHA-HBe4 cells were considered potential binding partners of p22 and were further analyzed based on their intensity and confidence scores.

### AlphaFold3 modelling of the C1QBP-p22 interaction

Models of the C1QBP in complex with p22 were performed on the AlphaFold3 server (https://alphafoldserver.com/) [106]. Both full-length p22 and the CTD were used in prediction runs. The accuracy of the predicted relative positions of the subunits within the complex interface is measured by the predicted template modeling (ipTM). ipTM values higher than 0.8 represent confident high-quality predictions. In this study, predication run without the NTD of p22 (CTD + C1QBP) increased ipTM from 0.62 to 0.8. Structural representation and electrostatic potential analysis with performed using PyMOL 2.5.2 with the APBS Electrostatics plugin.

### Statistical analysis

Quantitative data is presented as the mean ± standard deviation (SD). Graphs were generated using GraphPad Prism 10.

## Supporting information

S1 Fig Subcellular localization of C1QBP and dMTS. (A) HepG2 cells were transfected with either a control vector, C1QBP, or dMTS. Three days post-transfection, cells were harvested and fractionated to separate cytosolic and mitochondrial fractions, with unfractionated cells used as input controls. Western blot analysis of C1QBP and dMTS was performed on input and fractionated samples, using GAPDH as a cytosolic marker and VDAC as a mitochondrial marker, respectively. β-actin served as a loading control for input samples. (B) HepG2 cells were transfected with the control vector, C1QBP-FLAG, or dMTS-FLAG for 3 days. Cells were harvested and processed as described in panel A, and Western blot analysis was performed for C1QBP-FLAG and dMTS-FLAG using an anti-FLAG antibody.
(TIF)

S2 Fig Further validation of p22-C1QBP interaction and mapping the p22-interacting domain on C1QBP. (A) HepHA-HBe4 cells were transfected with the control vector, full-length C1QBP-FLAG, or each indicated C1QBP-FLAG truncation mutant (1–160-FLAG, 1–220-FLAG, 74–160-FLAG, and 74–220-FLAG). Three days post-transfection, cells were subjected to co-IP using anti-HBc antibody. Input and co-IP samples were analyzed by Western blot using anti-FLAG and anti-HA antibodies, with β-actin serving as a loading control for the input samples. (B) HepG2 cells were co-transfected with PC and either a control vector, C1QBP-FLAG, or dMTS-FLAG. Three days post-transfection, cells were collected and processed as described in panel A, and Western blot was performed using anti-FLAG and anti-HBc antibodies.
(TIF)

S3 Fig AlphaFold3 modeling of the C1QBP-p22 complex. (A) AlphaFold3 models of C1QBP in complex with full-length p22 (left) and p22 CTD (middle). Both models show that the C-terminal end of the p22 CTD folds into a dipper-like domain and binds to the donut hole in the middle of trimeric C1QBP. The protomers of C1QBP are colored in light blue, light green, and pale cyan, respectively. Full-length p22 and CTD fragment are colored in magenta and orange, respectively. In AlphaFold3, ipTM value measures the accuracy of the predicted relative positions of the subunits within the complex. Values higher than 0.8 represent confident high-quality predictions. In this study, removing the p22 N-terminal domain (NTD) from the modelling of C1QBP complex increases ipTM value from 0.62 to 0.80. The model reveals that CTD of p22 interacts with the inner wall of the donut hole formed by aa 74–160 of C1QBP (right), consistent with results in Figs 2–3. (B) Comparison of AlphaFold3 models of p22 alone or in complex with C1QBP. CTD in p22 modelled alone does not show ordered structure, while those in complex with C1QBP adopt a triangular dipper-like fold in the C-terminal arginine-rich region. (C) Electrostatic potential surfaces of C1QBP and p22. The negatively and positively charged regions are indicated by red and blue, respectively (range: -5 to +5 kT/e). Highly negatively charged C1QBP (left) most likely interacts with the positively charged arginine-rich CTD of p22 through salt bridges and other interactions (middle). The triangular dipper-like domain of p22 CTD cluster arginines together to form a highly positively charged surface (right).
(TIF)

S4 Fig Subcellular localization of endogenous C1QBP in different cell lines. Immunofluorescence assay was performed on HepG2, A549, and HEK293T cells using MitoTracker (red), anti-C1QBP antibody (green), and nuclear dye DAPI (blue). The merged images show the colocalization of C1QBP and mitochondria, indicated by the yellow signal.
(TIF)

S5 Fig C1QBP inhibits HBV capsid and nucleocapsid formation without affecting viral transcription. (A) HepG2 cells were pre-treated with 10 μM of lamivudine (3TC) for 24 h and then transfected with pHBV1.3 and either a control vector or C1QBP-FLAG for an additional 5 days with continuous 3TC treatment. HBV total RNA and encapsidated pgRNA were analyzed by Northern blot. Western blot was performed to detect C1QBP-FLAG, p22, and HBc, with β-actin serving as a loading control. The cytoplasmic capsid was detected by capsid gel assay. (B) HepG2 cells were transfected with pHBV1.3 and either a control vector, C1QBP-FLAG, or dMTS-FLAG for 5 days. HBV total RNA, proteins (C1QBP-FLAG dMTS-FLAG, p22, HBc, and β-actin), cytoplasmic HBV capsid, and encapsidated pgRNA were analyzed as described in panel A. Cytoplasmic HBV core DNA was analyzed by Southern blot. (C) HepG2 cells were co-transfected with HBV enhancer II and core promoter-driven firefly luciferase reporter plasmid EnII/Cp-Luc and CMV-IE promoter-driven *Renilla* luciferase control reporter plasmid pRL-CMV, plus control vector, C1QBP-FLAG, or dMTS-FLAG for 3 days. Firefly luciferase activities were measured and normalized to *Renilla* luciferase signals. The relative luciferase activities were plotted as fold changes against the control group (mean ± SD, n = 3; ns: not significant).
(TIF)

S6 Fig Validation of HBc-p22-C1QBP interaction by co-IP. (A) HBc-p22 interaction. HepG2 cells were transfected with HA-PC along with either a control vector or HBc expression vector. Three days post-transfection, 10% of the cells were collected as input, while the remaining cells were subjected to co-IP using anti-HA antibody. Input and co-IP samples were analyzed by Western blot using anti-HA and anti-HBc antibodies. β-actin was used as a loading control for input samples. (B) HBc-p22-C1QBP interaction. HepG2 cells were co-transfected with dMTS-FLAG together with either a control vector, HBc, PC, or a combination of PC and HBc. Three days later, cells were subjected to co-IP assay using anti-FLAG antibody. The input and co-IP samples were analyzed by Western blot using anti-HBc and anti-FLAG antibodies.
(TIF)

S7 Fig The centrifugal transfection efficiency in PHHs. PHHs were transfected with an eGFP vector using Lipofectamine 3000. To enhance transfection efficiency, the cell culture plates were centrifuged at 1,000 rpm for 15 min at RT after adding the transfection mixture. After 3 days of culture, transfection efficiency was assessed by detecting eGFP expression under a fluorescence microscope.
(TIF)

S1 Data Source data for bar plots in this paper. (XLSX)
