# Supplementary figures and images for: HBV p22-interacting protein C1QBP inhibits viral replication through impeding nucleocapsid formation and nuclear import
Source: PLoS Pathog. 2025 Oct 17;21(10):e1013581. doi: 10.1371/journal.ppat.1013581 (PMC12533850; doi:10.1371/journal.ppat.1013581)

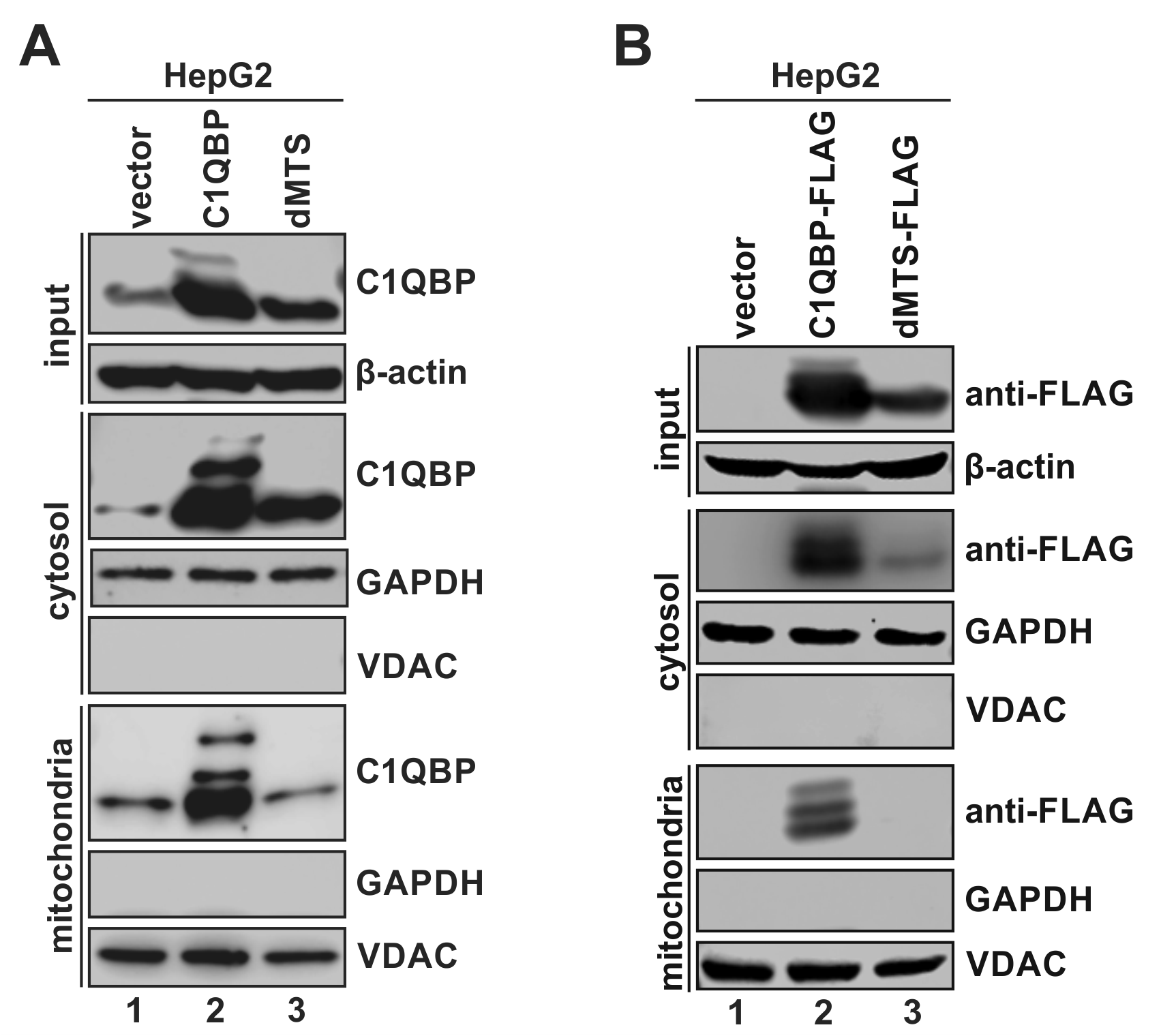

Supplement: S1 Fig — (A) HepG2 cells were transfected with either a control vector, C1QBP, or dMTS. Three days post-transfection, cells were harvested and fractionated to separate cytosolic and mitochondrial fractions, with unfractionated cells used as input controls. Western blot analysis of C1QBP and dMTS was performed on input and fractionated samples, using GAPDH as a cytosolic marker and VDAC as a mitochondrial marker, respectively. β-actin served as a loading control for input samples. (B) HepG2 cells were transfected with the control vector, C1QBP-FLAG, or dMTS-FLAG for 3 days. Cells were harvested and processed as described in panel A, and Western blot analysis was performed for C1QBP-FLAG and dMTS-FLAG using an anti-FLAG antibody. (TIF) [file ppat.1013581.s001.tif]

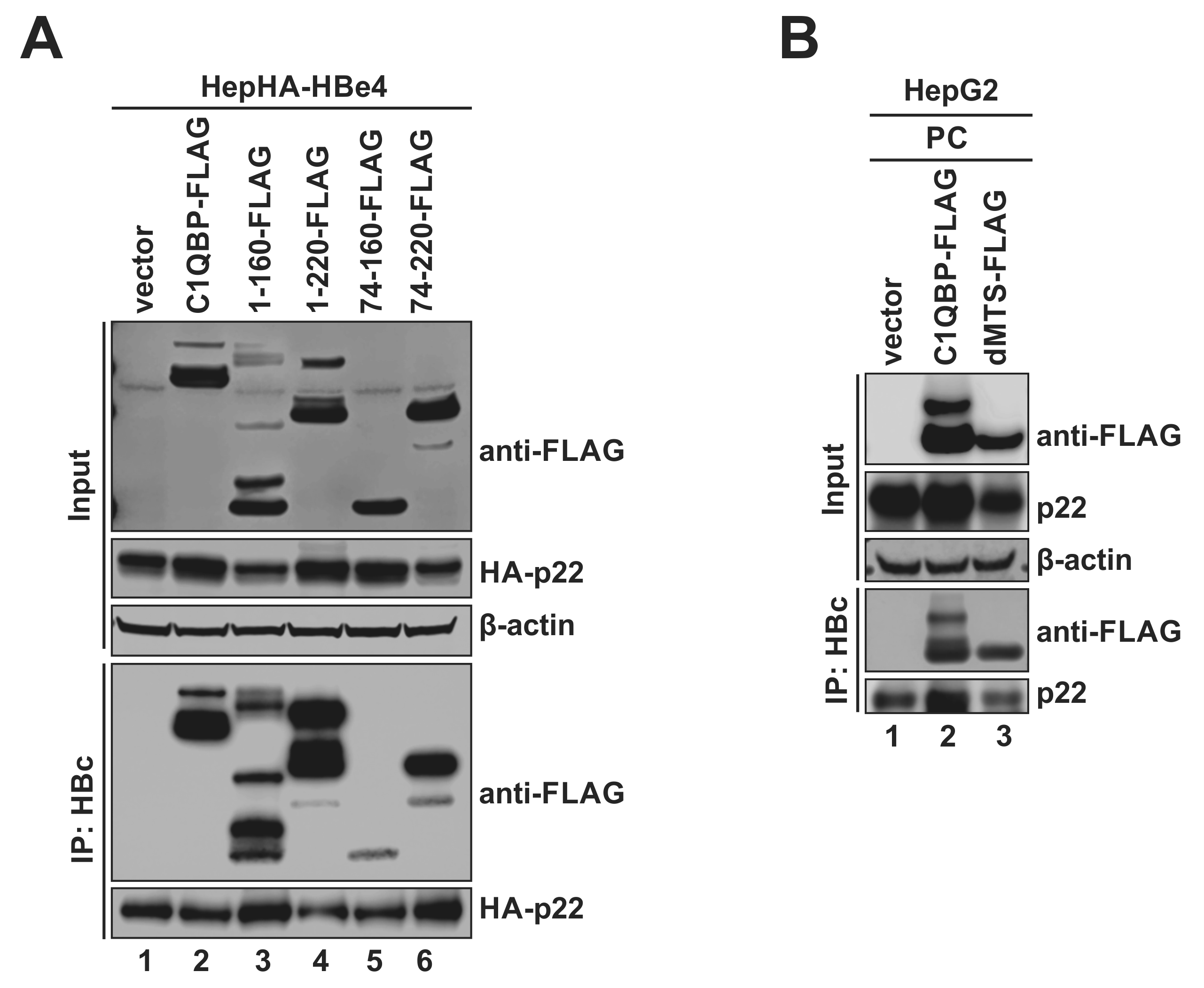

Supplement: S2 Fig — (A) HepHA-HBe4 cells were transfected with the control vector, full-length C1QBP-FLAG, or each indicated C1QBP-FLAG truncation mutant (1–160-FLAG, 1–220-FLAG, 74–160-FLAG, and 74–220-FLAG). Three days post-transfection, cells were subjected to co-IP using anti-HBc antibody. Input and co-IP samples were analyzed by Western blot using anti-FLAG and anti-HA antibodies, with β-actin serving as a loading control for the input samples. (B) HepG2 cells were co-transfected with PC and either a control vector, C1QBP-FLAG, or dMTS-FLAG. Three days post-transfection, cells were collected and processed as described in panel A, and Western blot was performed using anti-FLAG and anti-HBc antibodies. (TIF) [file ppat.1013581.s002.tif]

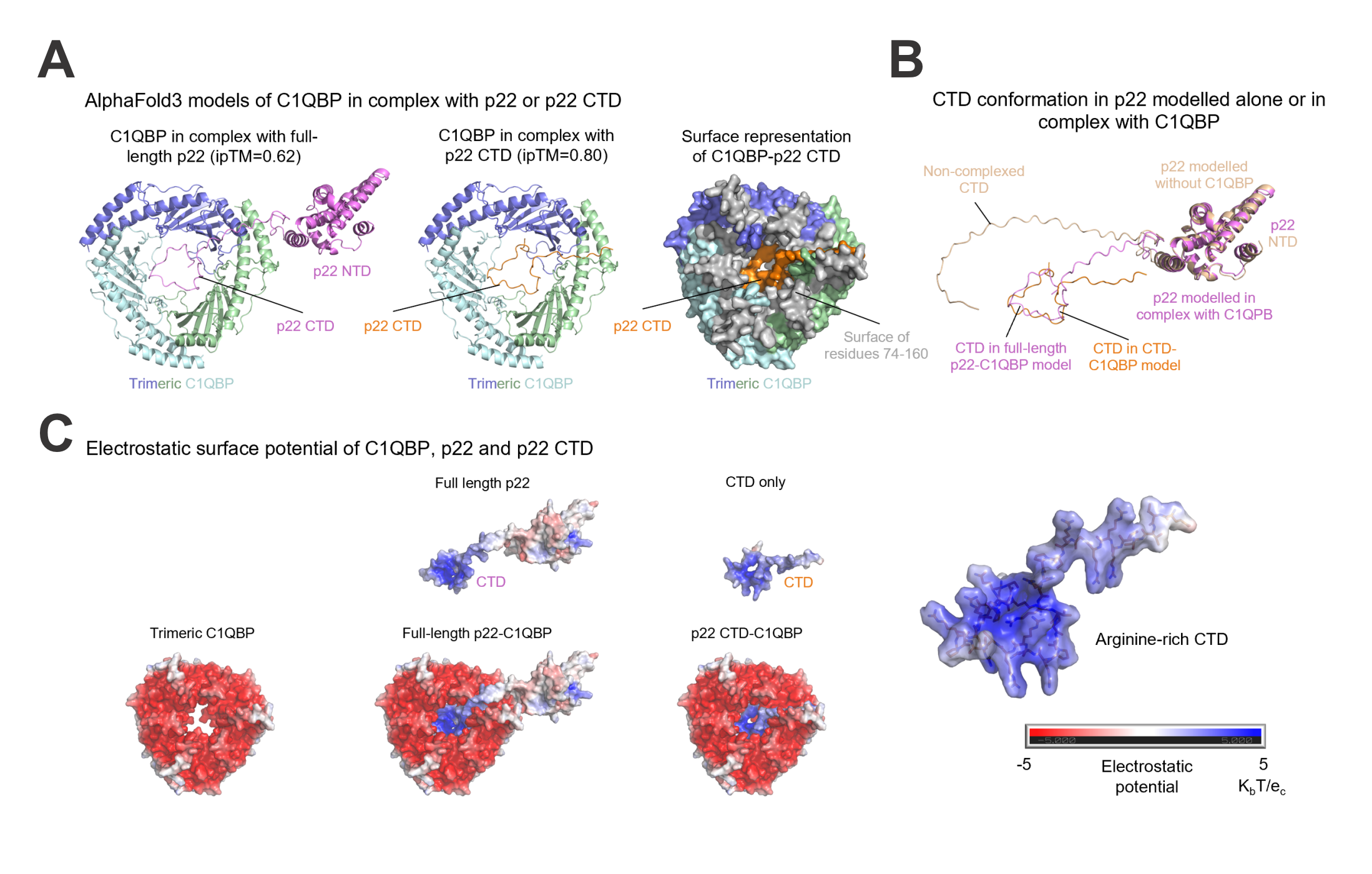

Supplement: S3 Fig — (A) AlphaFold3 models of C1QBP in complex with full-length p22 (left) and p22 CTD (middle). Both models show that the C-terminal end of the p22 CTD folds into a dipper-like domain and binds to the donut hole in the middle of trimeric C1QBP. The protomers of C1QBP are colored in light blue, light green, and pale cyan, respectively. Full-length p22 and CTD fragment are colored in magenta and orange, respectively. In AlphaFold3, ipTM value measures the accuracy of the predicted relative positions of the subunits within the complex. Values higher than 0.8 represent confident high-quality predictions. In this study, removing the p22 N-terminal domain (NTD) from the modelling of C1QBP complex increases ipTM value from 0.62 to 0.80. The model reveals that CTD of p22 interacts with the inner wall of the donut hole formed by aa 74–160 of C1QBP (right), consistent with results in Figs 2–3. (B) Comparison of AlphaFold3 models of p22 alone or in complex with C1QBP. CTD in p22 modelled alone does not show ordered structure, while those in complex with C1QBP adopt a triangular dipper-like fold in the C-terminal arginine-rich region. (C) Electrostatic potential surfaces of C1QBP and p22. The negatively and positively charged regions are indicated by red and blue, respectively (range: -5 to +5 kT/e). Highly negatively charged C1QBP (left) most likely interacts with the positively charged arginine-rich CTD of p22 through salt bridges and other interactions (middle). The triangular dipper-like domain of p22 CTD cluster arginines together to form a highly positively charged surface (right). (TIF) [file ppat.1013581.s003.tif]

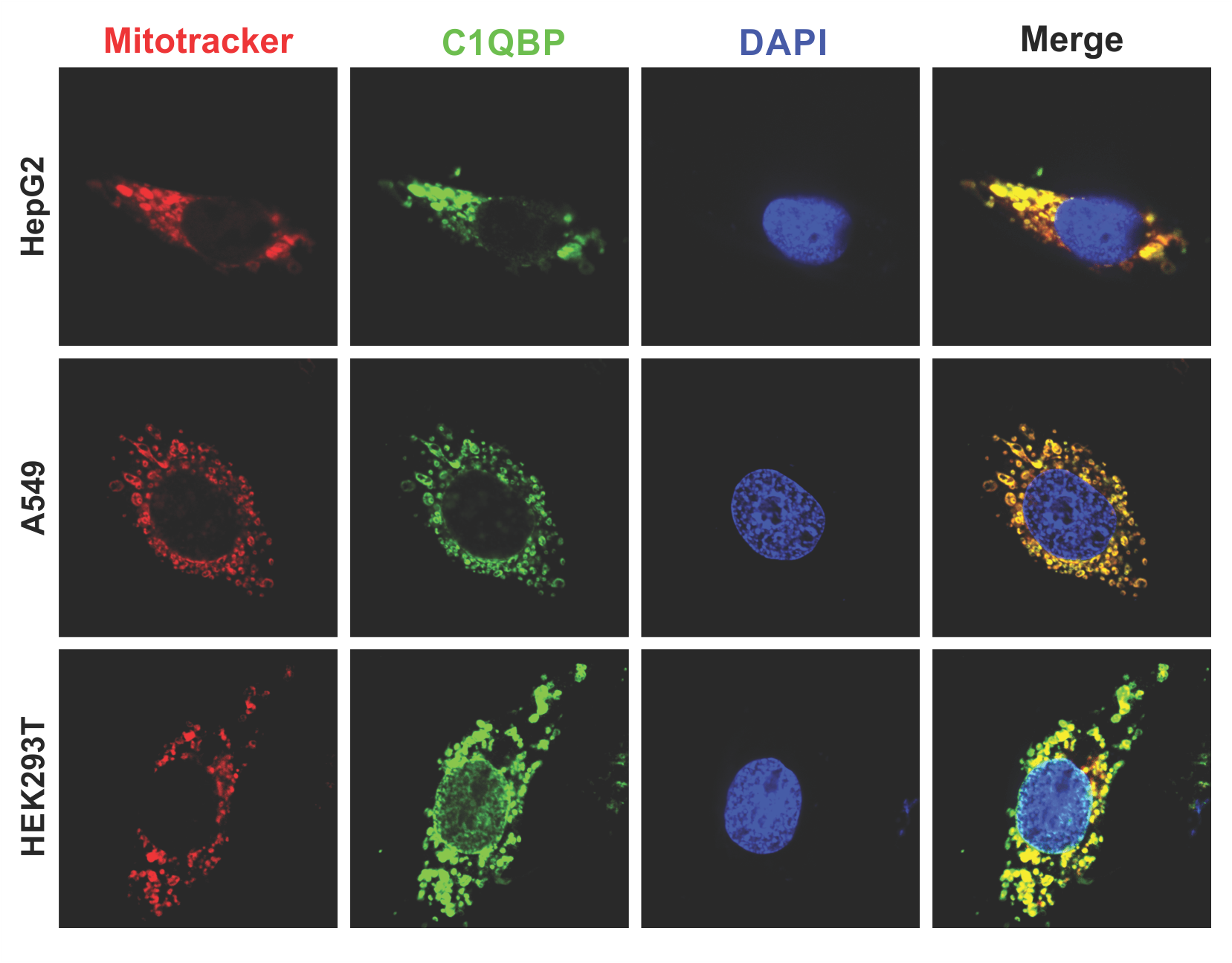

Supplement: S4 Fig — Immunofluorescence assay was performed on HepG2, A549, and HEK293T cells using MitoTracker (red), anti-C1QBP antibody (green), and nuclear dye DAPI (blue). The merged images show the colocalization of C1QBP and mitochondria, indicated by the yellow signal. (TIF) [file ppat.1013581.s004.tif]

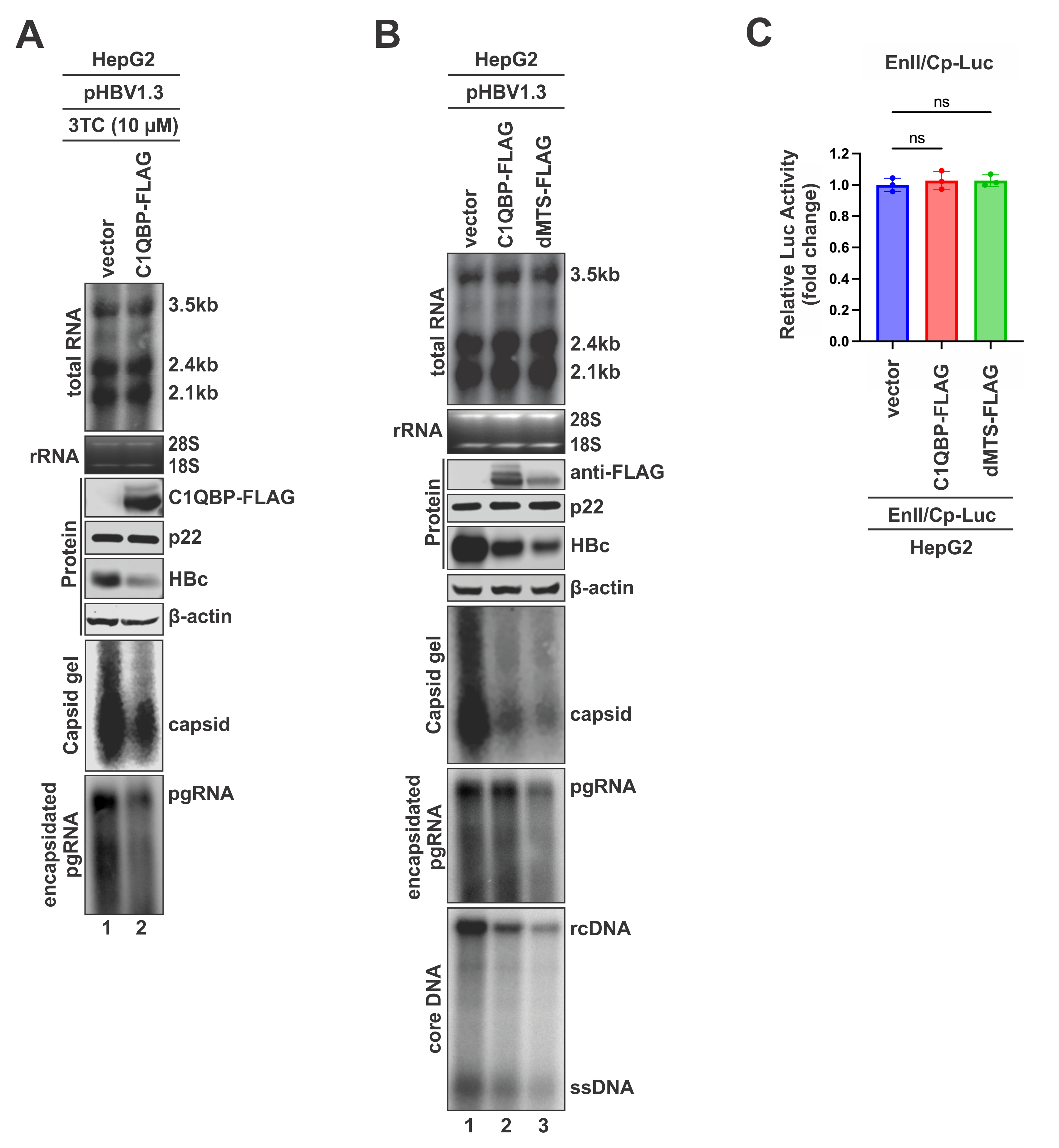

Supplement: S5 Fig — (A) HepG2 cells were pre-treated with 10 μM of lamivudine (3TC) for 24 h and then transfected with pHBV1.3 and either a control vector or C1QBP-FLAG for an additional 5 days with continuous 3TC treatment. HBV total RNA and encapsidated pgRNA were analyzed by Northern blot. Western blot was performed to detect C1QBP-FLAG, p22, and HBc, with β-actin serving as a loading control. The cytoplasmic capsid was detected by capsid gel assay. (B) HepG2 cells were transfected with pHBV1.3 and either a control vector, C1QBP-FLAG, or dMTS-FLAG for 5 days. HBV total RNA, proteins (C1QBP-FLAG dMTS-FLAG, p22, HBc, and β-actin), cytoplasmic HBV capsid, and encapsidated pgRNA were analyzed as described in panel A. Cytoplasmic HBV core DNA was analyzed by Southern blot. (C) HepG2 cells were co-transfected with HBV enhancer II and core promoter-driven firefly luciferase reporter plasmid EnII/Cp-Luc and CMV-IE promoter-driven Renilla luciferase control reporter plasmid pRL-CMV, plus control vector, C1QBP-FLAG, or dMTS-FLAG for 3 days. Firefly luciferase activities were measured and normalized to Renilla luciferase signals. The relative luciferase activities were plotted as fold changes against the control group (mean ± SD, n = 3; ns: not significant). (TIF) [file ppat.1013581.s005.tif]

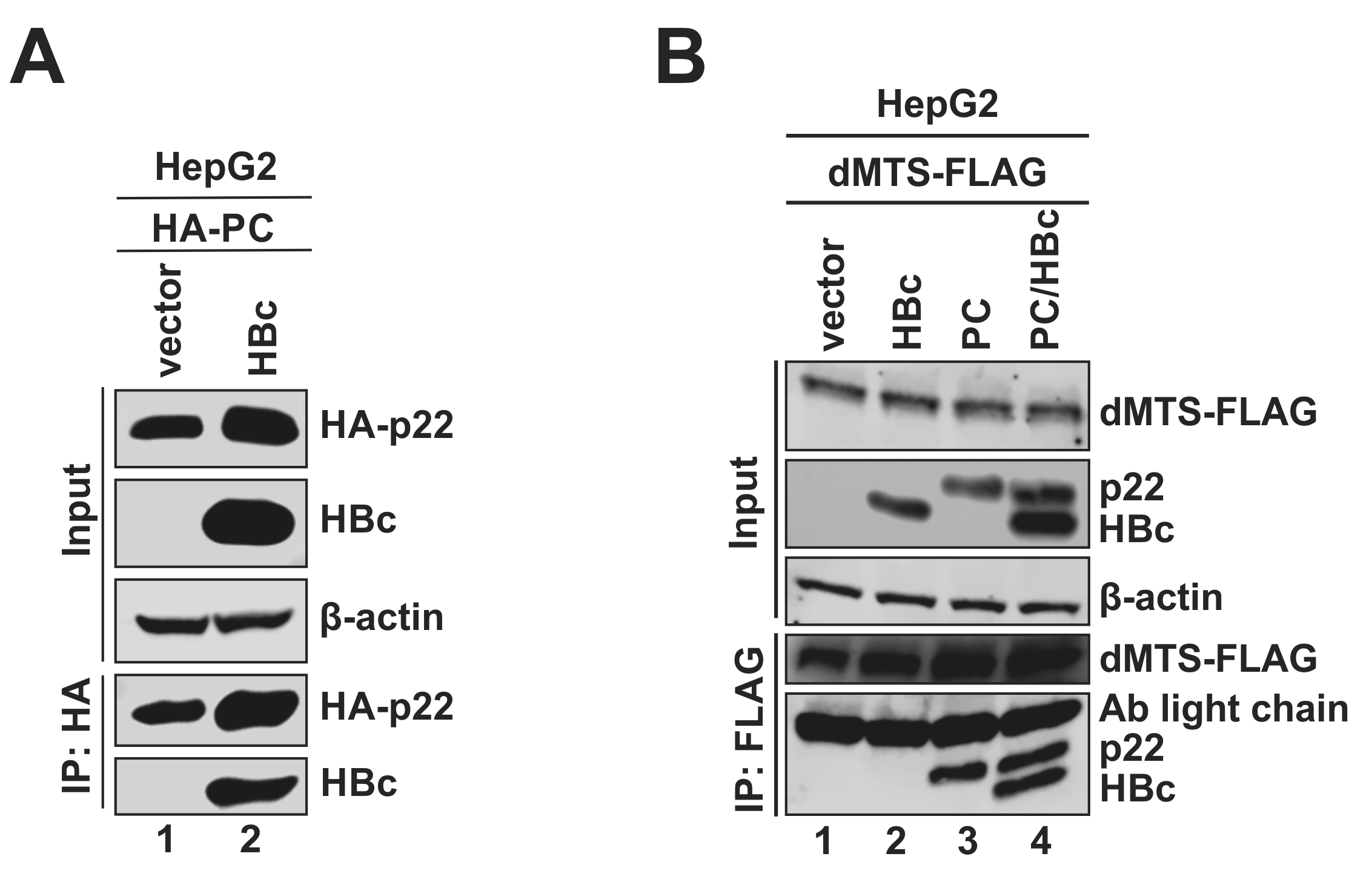

Supplement: S6 Fig — (A) HBc-p22 interaction. HepG2 cells were transfected with HA-PC along with either a control vector or HBc expression vector. Three days post-transfection, 10% of the cells were collected as input, while the remaining cells were subjected to co-IP using anti-HA antibody. Input and co-IP samples were analyzed by Western blot using anti-HA and anti-HBc antibodies. β-actin was used as a loading control for input samples. (B) HBc-p22-C1QBP interaction. HepG2 cells were co-transfected with dMTS-FLAG together with either a control vector, HBc, PC, or a combination of PC and HBc. Three days later, cells were subjected to co-IP assay using anti-FLAG antibody. The input and co-IP samples were analyzed by Western blot using anti-HBc and anti-FLAG antibodies. (TIF) [file ppat.1013581.s006.tif]

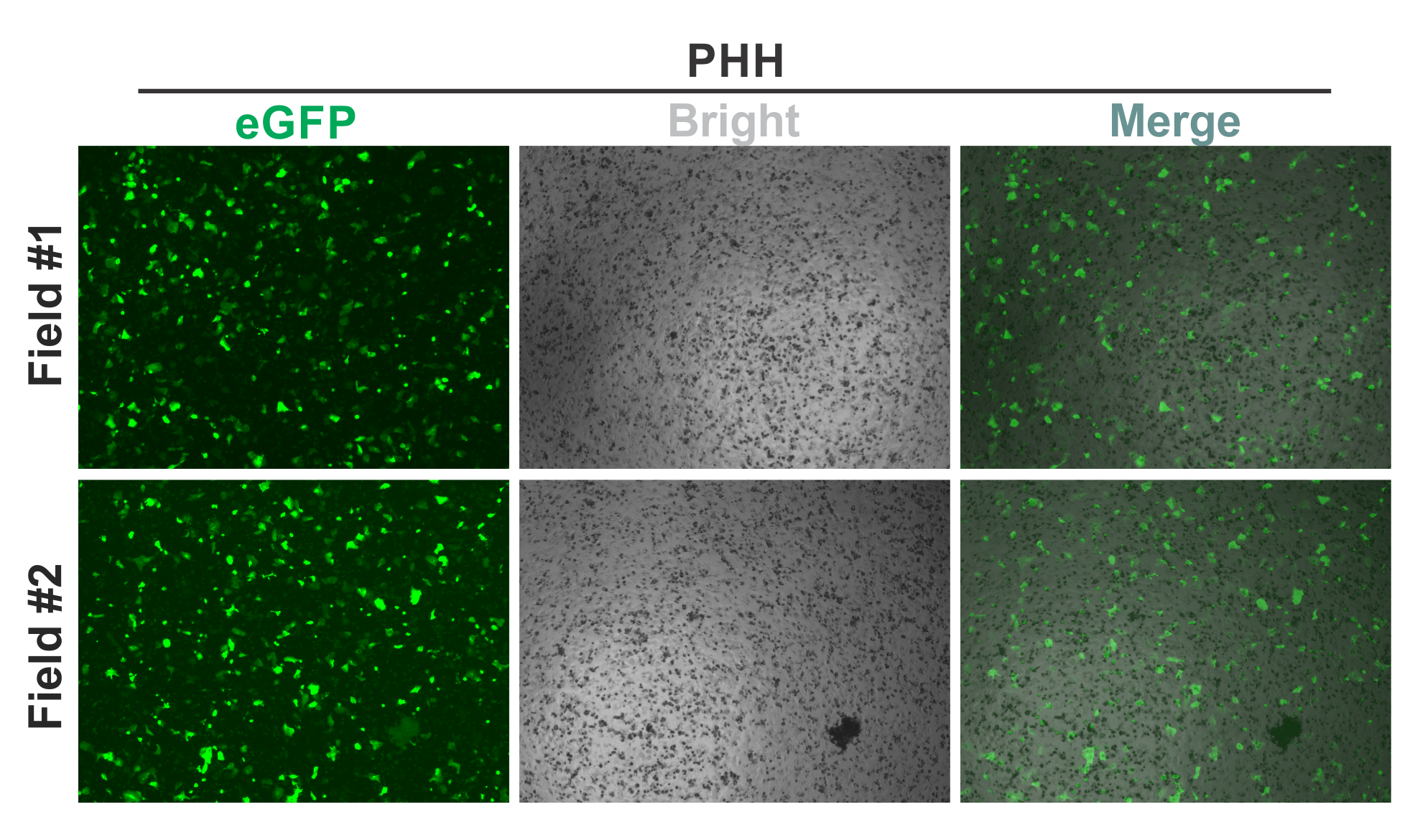

Supplement: S7 Fig — PHHs were transfected with an eGFP vector using Lipofectamine 3000. To enhance transfection efficiency, the cell culture plates were centrifuged at 1,000 rpm for 15 min at RT after adding the transfection mixture. After 3 days of culture, transfection efficiency was assessed by detecting eGFP expression under a fluorescence microscope. (TIF) [file ppat.1013581.s007.tif]
